# Personality-based pair programming: toward intrinsic motivation alignment in very small entities

**DOI:** 10.7717/peerj-cs.2774

**Published:** 2025-04-01

**Authors:** Marcel Valovy, Alena Buchalcevova

**Affiliations:** Department of Information Technology, University of Economics Prague, Prague, Czech Republic

**Keywords:** Intrinsic motivation, Agile software development, Linear mixed-effects, Software engineering, Mixed-methods research, Big Five, Large language models (LLMs), Pair programming, ISO/IEC 29110, Inter-coder reliability

## Abstract

**Aim:**

This study explores whether personality‐based role assignments (Pilot, Navigator, Solo) can raise intrinsic motivation in pair programming, focusing on designing a framework and process extension for the resource‐constrained environment of very small entities (VSEs).

**Method:**

We employed a mixed‐methods design across three quasi-experimental datasets (*n* = 73 participants), applying linear mixed‐effects (LME) modeling to assess motivational outcomes and thematically analyzing (*n* = 25) interviews for socio‐psychological insights.

**Findings:**

Openness strongly correlates with Pilot roles; Extraversion & Agreeableness favor Navigator roles; and Neuroticism aligns more comfortably with Solo roles—each yielding substantial boosts in intrinsic motivation (up to 60–65%). Twelve qualitative themes underscore the influence of mentorship, pairing constellations, and flow disruptions on developer experiences.

**Implications:**

Building on these results, we propose the role‐optimization motivation alignment (ROMA) framework, mapped to the ISO/IEC 29110 Software Basic Profile and Agile Guidelines, with practical tasks (T1–T7) to facilitate systematic role–trait alignments in small agile teams. Although our data primarily involve Gen‐Z undergraduates, the recurring patterns suggest broader applicability, further supported by a separately published application for ongoing generalizability.

**Conclusion:**

Personality‐driven role optimization may significantly enhance collaboration and developer satisfaction in VSEs, though further studies in professional settings and investigations into AI‐assisted or distributed pair programming are warranted.

## Introduction

The global software ecosystem relies heavily on very small entities (VSEs)—small teams operating with stringent resource constraints, evolving customer demands, and limited process maturity (Laporte et al., 2018). While Agile methods can streamline processes, many VSEs grapple with human factors that influence software quality and sustainability (O’Connor & Laporte, 2014; Lenberg, Feldt & Wallgren, 2015). Among these factors, intrinsic motivation plays a pivotal role. Developers who derive inherent satisfaction from their work tend to be more engaged, creative, and resilient, and this principle extends to other resource-constrained contexts—such as small/home-offices (SOHOs)—which increasingly employ artificial intelligence (AI)-driven development tools to optimize workflow efficiency (cf. Schleiger et al., 2024).

A key driver of intrinsic motivation lies in the interplay between individual personality traits and basic psychological needs. Drawing on Self-Determination Theory and the Big Five personality model, we recognize that developers vary in how they experience autonomy, competence, and relatedness—factors that drive sustained interest and well-being at work (Ryan & Deci, 2000, 2017; McCrae & Costa, 2008). Traditional team configurations often overlook these differences, potentially undermining motivation and hindering performance.

Pair programming is a case in point. While widely promoted for its benefits in code quality, knowledge transfer, and collaboration, pair programming typically applies a one-size-fits-all approach to role assignment. By contrast, if roles like Pilot and Navigator were aligned with personality traits, pair programming might become more intrinsically motivating and effective. Such a refined approach could be particularly transformative for VSEs, where each contributor’s performance and satisfaction carry outsized importance due to the team’s small scale. Therefore, this study aims to address three objectives:

**O1:** Identify and analyze personality-based preferences for pair programming roles.
**O2:** Develop the Role-Optimization Motivation Alignment (ROMA) framework to enhance intrinsic motivation and team productivity among software professionals in VSEs, SOHOs, and undergraduate courses.
**O3:** Extend the ISO/IEC 29110 Software Basic Profile and Agile Guidelines and outline a distributed personality-based pair programming application for the continuous generalization and adaptation of the findings in these contexts.

This study explores how personality-based role assignments in pair programming can enhance intrinsic motivation, ultimately guiding VSEs to optimize programming roles by aligning their developer traits and psychological needs. Using a mixed-methods approach with Gen-Z undergraduates—future professionals whose skills and motivations can, under controlled conditions, approximate those of novice practitioners (Falessi et al., 2018; Feldt et al., 2018)—we integrate quantitative and qualitative data from multiple studies to:

- map personality-driven programming role preferences,
- uncover socio-psychological and group-dynamic effects, and
- propose strategies for implementing these insights in compliance with the *ISO/IEC 29110 Software Basic Profile* and associated *Agile Guidelines*.

Based on those objectives, we formulated three research questions that guide our study:

**RQ1:** What new understanding of personality-based motivational preferences for pilot, navigator, and solo programming roles can be achieved?
**RQ2:** What are the socio-psychological and group dynamic effects of pair programming?
**RQ3:** How should the ROMA framework, optimizing programming roles and their assignments to increase individual motivation and team productivity, be utilized in VSEs?

Motivated to bridge the gap between social psychology and software engineering practice and to solve the challenge of optimizing programming roles to increase intrinsic motivation, this research lays a theoretical and practical foundation for cultivating more fulfilling, high-performing development processes in VSEs and other resource-constrained teams, including AI-supported SOHOs. In the following sections, we situate this work in the broader body of literature (Background), describe our methodology (Materials & Methods), present our cross-analytical, mixed-methods findings (Empirical Findings), and detail how the ROMA framework and related artifacts can be applied to improve intrinsic motivation and team collaboration in small-scale software development settings (Personality-Based Pair Programming Artifacts). Finally, we discuss the validity of our results and implications for future research (Discussion & Conclusion).

## Background

This section reviews key constructs and theoretical underpinnings that inform this study. We examine pair programming in the context of VSEs and undergraduate software engineering settings, integrate perspectives from personality psychology and motivation research, and relate these insights to the ISO/IEC 29110 standard series and its Software Basic Profile and Agile Guidelines. This interdisciplinary backdrop supports the study’s hypotheses regarding the relationship between personality traits, role preferences, and intrinsic motivation.

### Pair programming and variations across contexts

Pair programming emerged as a collaborative technique in which two developers—traditionally a Pilot (driver) and a Navigator (observer)—work together at a single workstation (Williams & Kessler, 2003). This approach often enhances code quality, fosters knowledge transfer, and accelerates learning (Plonka et al., 2015). Over time, variations have arisen, including remote pair programming, promiscuous pairing (frequent partner rotation), and mob programming (involving larger groups), each reflecting efforts to optimize collaboration (Smite et al., 2021; Zieris & Prechelt, 2020a; Belshee, 2005; Zuill & Meadows, 2014).

In educational environments, pair programming helps students learn from peers, gain problem-solving strategies, and build communication skills (Tan, Wu & Shanshan, 2023). However, evidence for universal benefits remains mixed, partly due to publication bias and differing task complexities (Hannay et al., 2009a; Bowman et al., 2020). While some tasks are better suited to pair work, others may not yield the same productivity or quality gains (Arisholm et al., 2007).

For VSEs, where human resources are limited and each team member’s productivity is critical, pair programming could be particularly impactful. Yet, current practices typically assume a uniform approach to role assignment, overlooking how individual traits might influence motivation and effectiveness (Hannay et al., 2009b; Aghaee et al., 2015; Demir & Seferoglu, 2021).

### Personality, motivation, and behavioral software engineering

Motivation in software engineering has begun to draw attention within the growing field of behavioral software engineering, which emphasizes human factors influencing software quality and team performance (Lenberg, Feldt & Wallgren, 2015). Personality traits—relatively stable patterns of thought, emotion, and behavior—play a key role in shaping motivational processes (Corr, DeYoung & McNaughton, 2013; DiDomenico & Ryan, 2017; Dweck, 2017). The *Big Five* personality model (Openness, Conscientiousness, Extraversion, Agreeableness, Neuroticism) provides a widely accepted framework for investigating how individual differences might affect teamwork, role preferences, and engagement in software tasks (McCrae & Costa, 2008; Cruz, da Silva & Capretz, 2015; Felipe et al., 2023).

Motivation itself can be understood through Self-Determination Theory (SDT), which posits that fulfilling basic psychological needs—competence, autonomy, and relatedness—fosters intrinsic motivation, leading to enhanced well-being, creativity, and sustained effort (Ryan & Deci, 2000, 2017). In software engineering, aligning tasks and roles with developers’ personalities and intrinsic motivations can potentially boost happiness, analytical problem-solving performance, creativity, satisfaction, and learning (Graziotin, Wang & Abrahamsson, 2014; Kuusinen et al., 2016).

### ISO/IEC 29110: a tailored standard series with Agile adaptations

The ISO/IEC 29110 standard series was developed to help VSEs adopt industry-recognized software processes without overwhelming them with unnecessary complexity (Laporte et al., 2018). Organized into progressive profiles—Entry, Basic, Intermediate, and Advanced—it offers a stepwise approach. The Software Basic Profile, for instance, targets small projects with relatively straightforward, non-mission-critical conditions, providing structured guidance on planning, execution, verification, and delivery (ISO/IEC DIS 29110-5-1-2, 2023).

To make the Basic Profile more agile and responsive to market demands, the ISO/IEC JTC1/SC7 Working Group 24 introduced Agile Software Development Guidelines (Galván-Cruz et al., 2021; ISO/IEC DIS 29110-5-4, 2024). These guidelines integrate familiar agile events: a project vision meeting sets initial priorities, an estimation meeting refines the workload, sprint planning and execution structure iterative development cycles, daily scrums coordinate tasks, and sprint reviews and retrospectives encourage continuous improvement. By mapping these agile practices onto the Basic Profile and clarifying the roles of Customer, Product Owner, Scrum Master, and Development Team, the guidelines bridge the gap between formal process definitions and the adaptive, people-oriented ethos of agile methods (Beck et al., 2001; Abrahamsson et al., 2017; O’Connor & Laporte, 2014).

Yet, even with these refinements, ISO/IEC 29110 and the associated guidelines do not explicitly address the human factors that influence motivation and productivity. Personality-based pair programming role assignments could enrich this framework, allowing VSEs to account for intrinsic motivation when defining roles and responsibilities. In doing so, these small organizations may better harness their limited human capital, improving both team satisfaction and software quality.

### Research hypotheses on personality-role alignments

Based on research questions RQ1 & RQ2, we formulated six hypotheses before data collection. Building on the literature linking personality traits, motivation, and performance (Barrick, Stewart & Piotrowski, 2002; Schmitt et al., 2003; DeYoung, 2015), as well as the growing interest in personality-based factors in pair programming (Salleh, Mendes & Grundy, 2011, 2014; Demir & Seferoglu, 2021) and our previous work on personality’s effect on work motivation in different software engineering roles (Valový, 2022), these hypotheses reflect a vision that carefully matching personality traits to development roles can improve intrinsic motivation and performance, and potentially guide future integrations with artificial intelligence (AI)-assisted coding tools (Valový & Buchalcevova, 2023).

*H1 and H1-Cor: Intrinsic motivation varies by Pilot, Navigator, and Solo roles, and this variation remains stable across individuals*. Prior studies suggest that splitting software engineering tasks into distinct roles alters team dynamics and may variably affect motivation (Brooks, 1987; Chong & Hurlbutt, 2007; Sfetsos et al., 2009; Williams & Kessler, 2003).

*H2 and H3: Software engineers exhibit distinct personality types, and these personality types prefer different pair programming roles*. Given that personality traits can form characteristic clusters due to evolutionary factors (Riemann & Kandler, 2010; Nettle, 2006; Penke, Denissen & Miller, 2007), certain trait constellations are hypothesized to align with specific role preferences (DiDomenico & Ryan, 2017; Dweck, 2017; Ryan & Deci, 2017).

*H4: Individuals with high openness to experience prefer the Pilot role*. The Pilot’s tasks often demand creative ideation and rapid adaptation. Given that openness is associated with curiosity, divergent thinking, and the pursuit of novel solutions, we predict that those high in openness will prefer the role responsible for tactical code construction and inventive problem-solving (DeYoung, 2015).

*H5: Individuals with high extraversion and agreeableness prefer the Navigator role*. Agreeableness supports smooth interpersonal interactions, while extraversion correlates with energetic engagement, sociability, and multitasking in teamwork, aligning with the Navigator’s strategic oversight and communicative responsibilities (Feldt et al., 2010).

*H6 and H6-Cor: Individuals with low extraversion and high neuroticism prefer the Solo role, and conversely, those with high extraversion and low neuroticism do not prefer Solo work*. High neuroticism reflects sensitivity to negative emotions and may drive a preference for solitary tasks with fewer social demands, while low extraversion similarly reduces the appeal of collaborative coding (Latham, 2012). Conversely, highly extraverted, emotionally stable developers may find introspective Solo programming less intrinsically motivating, favoring roles with more interpersonal engagement and emotional reward.

By empirically testing these predictions, we aim to contribute a more nuanced understanding of how individual differences can inform role assignments and potentially enhance intrinsic motivation and productivity in resource-constrained software development environments. In the future, these insights may also inform how human-AI pair programming could be structured to align with distinct personality traits.

## Materials and methods

This section details the philosophical stance, research design, participant selection, data collection procedures, and analytical methods of the present study. It explains the quantitative and qualitative approaches, the handling of repeated measures and personality-based analyses, and the process for establishing inter-coder reliability in the thematic analysis.

### Philosophical stance and research design

Our mixed-methods examination of personality-driven programming role assignments and their intrinsic motivation alignment is guided by critical realism, essentialism, and pragmatist axiology. Critically realist ontology posits that an objective reality exists, but our understanding of psychological constructs like personality and motivation is inherently partial and mediated by measurement and interpretation (Bhaskar, 1975). Essentialist epistemology complements this by treating constructs like personality traits and intrinsic motivation as having relatively stable, identifiable characteristics or “essences” that can be systematically explored and understood through reports on their ideation (Creswell & Creswell, 2022). Pragmatist axiology then serves to direct the creation and evaluation of artifacts toward ethically sound outcomes that address societal needs (Mbanaso, Abrahams & Okafor, 2023).

#### Research approach

The study combines deductive (theory-driven) and inductive (data-driven) approaches to theory development in a mixed-methods strategy (Johnson, Onwuegbuzie & Turner, 2007). Quantitative experiments, informed by established theories (*e.g*., Self-Determination Theory, Ryan & Deci, 2017; Big Five Personality Traits, McCrae & Costa, 2008), tested hypotheses about personality-role interactions. In contrast, an essentialist thematic analysis (Braun & Clarke, 2006) inductively derived themes from participant interviews without imposing preconceived frameworks. This approach enabled deeper insights into experiential aspects of role assignments and motivation, allowing emergent results to surface (Seaman, Hoda & Feldt, 2025).

By merging critical realism with essentialism, we aimed to identify cause-effect relationships in motivation and role assignments while also acknowledging that participants’ personal accounts reflect essential features of their psychological experiences.

#### Mixed-methods quasi-experimental design

Due to practical constraints, we adopted a quasi-experimental design with convenience sampling in a classroom setting of advanced undergraduate software engineering students at the Prague University of Economics and Business. This pragmatic design enabled us to investigate how personality traits interact with assigned programming roles (Pilot, Navigator, Solo) to influence intrinsic motivation. We integrated quantitative and qualitative data as follows:

1. *Quantitative phase:* Participants undertook controlled programming tasks under varying roles. We collected repeated measures of intrinsic motivation and personality profiles.
2. *Qualitative phase:* Semi-structured interviews explored participants’ subjective experiences, adding contextual depth to the statistical findings.
3. *Cross-study integration:* We compared and integrated new findings with previous research (Valový, 2023a, 2023b) to build a comprehensive understanding of motivational dynamics in pair programming.

#### Participants and ethical considerations

We drew on two cohorts of advanced undergraduate software engineering students: 40 participants from a Winter 2021 study (WS’21; Valový, 2023a) and 26 participants from a Summer 2022 study (SS’22: the current PeerJ dataset), totaling 66 experimental participants who completed all required sessions and were included in the quantitative analysis. For the qualitative interviews (phases 2 and 3), we recruited participants from three studies: twelve participants from the *WS’21* cohort, six participants from the *SS’22* cohort, and seven participants from a Winter 2022 study (WS’22; Valový, 2023b). This yielded a total of *25* interview participants. Counting these additional seven interviewees from *WS’22*, the overall pool across studies included *73* unique individuals (66 from the two main quantitative cohorts plus 7 from WS’22).

All studies were carried out under similar conditions in quasi-experimental settings with advanced undergraduate software engineering students at the Prague University of Economics and Business. Although these students are not seasoned professionals, prior empirical software engineering literature supports using advanced students as proxies for novice practitioners in controlled experimental conditions (Falessi et al., 2018; Feldt et al., 2018). This limitation is further discussed in the Limitations and Generalizability section.

In accordance with the ethical guidelines of the institutional review board, a consent waiver was obtained due to the minimal risks posed to participants and the educational context. Nevertheless, participants were fully informed about the nature of the study, and ethical principles, including anonymization of data and voluntary participation, were strictly followed.

### Quantitative phase: experimental design and measures

#### Experimental context and task design

The quasi-experiment took place in a classroom setting. Participants worked on 24 programming tasks across four sessions (six tasks per session). Tasks were designed to have similar complexity and duration, covering various areas of software engineering, such as creating graphical user interfaces for adventure games, implementing 2D animations in JavaFX, and developing multi-user Spring Boot web applications. Ensuring task comparability aimed to attribute observed motivational differences to role assignments rather than task difficulty variations.

We manipulated the key factor—assigned programming roles—while controlling confounding variables (*e.g*., task difficulty, time allocation) at a constant level (Wohlin et al., 2012). Across both control and test sessions, *a total of 1,092 intrinsic motivation inventories* were collected from 66 participants.

#### Roles, role rotation, and experimental conditions

Participants assumed three roles:

- *Pilot:* wrote the code and implemented solutions,
- *Navigator:* provided conceptual oversight, feedback, and high-level guidance,
- *Solo:* worked independently without a partner.

Regular role rotation at fixed intervals (every 10 min) ensured balanced exposure to all roles. Initially, participants paired with the students sitting next to them (convenience sampling). To address occasional absenteeism (four absences due to illness) and ensure continuous participation, we sometimes formed three-person groups. This group reconfiguration minimized missing data but introduced variability. By focusing on repeated measures at the individual level, we reduced the impact of these configurations on motivational comparisons.

#### Paired design

A pilot session familiarized participants with the roles and the psychometric measures. In the following two experimental sessions, participants were split into *control* (Solo only) and *test* (Pilot/Navigator rotation) groups. This arrangement with enforced rotations prevented “free-riding” and ensured balanced participation in both coding and reviewing tasks (Forsyth & Elliott, 1999; Gallis, Arisholm & Dyba, 2003).

In the final (fourth) session, all participants were moved into a test condition (Pilot/Navigator rotation), allowing each participant to experience all three roles—Solo, Pilot, and Navigator—in equal measure. This structure ensured that every participant had a comprehensive exposure to each role, enabling more reliable comparisons of intrinsic motivation across roles.

#### Instruments and data collection

Standardized psychological measures are increasingly recommended in empirical software engineering, aligning with our essentialist stance that treats personality and motivation as stable constructs amenable to empirical assessment (Graziotin et al., 2021; Feldt et al., 2010). We therefore used:

- *Personality traits:* The Big Five Inventory (BFI-10; Rammstedt & John, 2007) assessed Openness, Conscientiousness, Extraversion, Agreeableness, and Neuroticism. Though shorter scales can reduce interpretative depth (Felipe et al., 2023), the BFI-10 generally maintains acceptable validity (test–retest reliabilities ≈ 0.62–0.75, convergent validity ≥0.83 with BFI-44). Participants completed the BFI-10 once at the beginning of each session, and mean trait scores across sessions were computed to enhance stability and reduce transient fluctuations.
- *Intrinsic motivation:* The Interest/Enjoyment subscale of the Intrinsic Motivation Inventory (IMI; Ryan & Deci, 2024) measured participants’ intrinsic motivation at the end of each programming round (*six rounds per session*). This subscale is widely recognized for its robust psychometric properties and for capturing intrinsic motivation directly.
  (Note: The IMI ranges from 7 to 35 per round, reflecting the sum of 7 items on a 5-point Likert scale.)

All participants had at least B2 English proficiency, ensuring comprehension of the original instrument items. Data collection proceeded without task interruptions—if participants had not finished a previous task, they could continue working in the next round, with instructors available for assistance.

#### Repeated measures, nested data structures, and analytical approach

Each participant performed multiple programming rounds in each role, resulting in repeated measurements of intrinsic motivation. Consequently, the data had a nested structure: multiple observations (rounds) per participant. To address such non-independence and repeated-measures design, we employed linear mixed-effects (LME) models (Pinheiro & Bates, 2000) using R’s *nlme* package.

In these models, each participant received a random intercept to account for individual baseline differences in motivation. Fixed effects included Role (Pilot, Navigator, Solo) and continuous Big Five personality traits (scaled 1–10). Interaction terms (*e.g*., Role × Openness) tested whether specific traits moderated the motivational effects of each role. The LME approach properly addressed the hierarchical nature of the data and the within-subject correlations, ensuring more valid inferences.

#### Two LME model specifications

1. *Interaction model (traits as covariates):* The first model incorporated *Role* and the continuous Big Five *traits*, along with their interactions, to examine how individual differences in personality influenced intrinsic motivation across roles.
2. *Cluster model (personality-based clusters):* We also performed a simple cluster analysis on averaged trait scores to assign each participant to a dominant trait cluster *(O, C, E, A, or N)*. A second LME model replaced the continuous trait interactions with a *Role × PersonalityCluster* interaction, assessing distinct motivational patterns by personality cluster.

#### *Post-Hoc* comparisons

We used the *emmeans* package for *post-hoc* comparisons, estimating marginal means and pairwise contrasts. These clarified differences in predicted motivation among roles and trait (or cluster) configurations. Effect sizes (Cohen’s *d*) were computed *via* the *eff_size()* function in emmeans, referencing the model’s residual SD.

#### Experimental validity and control factors

We endeavored to control key factors like task difficulty and session length; however, some contextual variables—time of day (9:15–12:30 AM) or occasional three-person group reconfigurations due to absenteeism—were not fully constrained. Such factors may influence motivation and performance and are acknowledged as limitations. The primary manipulated factor was the assigned programming role. Rather than aiming for a fully isolated laboratory environment, our goal was a robust quasi-experimental design that minimized major confounds.

Tasks were carefully designed to maintain equal size, complexity, and duration (Sjøberg et al., 2002; Vanhanen & Lassenius, 2005). Participants were informed that no external rewards (*e.g*., course credits) were tied to their performance, maintaining focus on intrinsic motivation (Vinson & Singer, 2008). Although partner compatibility may matter (Katira et al., 2004; Williams et al., 2006), previous studies suggest minimal confounding from partner personality when roles are clearly defined. Additionally, by measuring motivation rather than performance, we reduced potential confounds of varying individual ability (Arisholm et al., 2007; Latham, 2012).

#### Hypothesis testing

The LME models tested main and interaction effects of roles and personality traits (or clusters) on intrinsic motivation. Our key hypotheses included:

- *H1 & H1-Cor:* Role-based differences in intrinsic motivation exist and remain stable within individuals.
- *H2 & H3:* Distinct personality configurations (clusters) align with role preferences.
- *H4–H6:* Specific traits (*e.g*., high Openness) differentially modulate motivation across roles.

All statistical analyses and visualizations used *R version 4.4.1* (with RStudio 2024.04.2). Scripts, anonymized datasets, and computed outputs are publicly available as Supplemental Material.

### Qualitative phase: interviews and thematic analysis

#### Interview procedures

Shortly after the final experimental session, participants in the Summer 2022 cohort were invited *via* email to partake in recorded semi-structured interviews (*n* = 6) *via* MS Teams. These interviews, led by the authors, aimed to unfold naturally and garner in-depth insights into the participants’ experiences and perspectives (Creswell & Creswell, 2022). They ranged from 20 min (Subject S1) to 34 min (Subject S6), featuring a structured protocol of ~25 open-ended questions spanning seven thematic areas, encouraging participants to reflect on their motivations, challenges, and perceptions regarding the three programming roles.

To distinguish sources across our three cohorts, we adopt the following notation:

- *I#* for participants from the *WS’21* data set (12 interviews),
- *P#* for participants from the *WS’22* data set (7 interviews),
- *S#* for participants in the *SS’22* data set (6 interviews).

Each study reached saturation with respect to its original research questions, yet new or revised topics in subsequent interview protocols allowed for emergent insights and further refinement of the thematic framework.

#### Qualitative thematic analysis and inter-coder reliability

We adopted a reflexive thematic analysis approach (TA; Braun & Clarke, 2006, 2019) from an essentialist perspective, allowing themes to emerge inductively from participants’ accounts rather than imposing predetermined categories. The essentialist stance assumes that participants’ descriptions directly convey stable underlying “essences” of their experiences (Creswell & Creswell, 2022). In contrast to the quantitative approach, which was theory-driven, our qualitative analysis was data-driven, focusing on participants’ subjective viewpoints about pair programming roles and how personality shapes intrinsic motivation.

Initially, *Coder 1* transcribed all six *SS’22* interviews using Descript (v67.1.1) and performed open coding in MAXQDA (v22.3.0). Approximately 70–80 codes were generated at this stage. For instance, we had a broad code like “Difficult personality interactions” that conflated various personality-related conflicts. Another example was “Time management issues,” which mixed positive and negative views on time constraints without differentiation.

#### Inter-coder reliability procedure and code refinement

To strengthen interpretive rigor, we applied an iterative inter-coder reliability (ICR) process (O’Connor & Joffe, 2020):

*1. Pilot coding:*

We selected two interviews (~33%) from *SS’22*, two (~17%) from *WS’21*, and two (~29%) from *WS’22* for the pilot phase. *Coder 2*, who was blind to Coder 1’s assignments, independently coded these interviews. The unit of coding was the sentence, and codes were applied in a mutually exclusive manner to maintain clarity in code boundaries.

*2. Refinement of codes:*

Discrepancies between coders prompted splitting or merging of codes. For example, the broad “Difficult personality interactions” code was replaced by more specific labels, such as *“Extraversion contrast leads to conflict” (I3, S2)* and *“High neuroticism restricts flow” (I4, P1)*, capturing distinct personality-related challenges more explicitly.

Similarly, codes like “Time-limits cause confusion,” “Participants ask for more time,” and “Desire to rotate after finishing tasks” were unified under *“Mixed perceptions of time limits” (P5, S2)*. After consensus discussions to improve discriminant capability by splitting or merging redundant codes, the set was reduced to *60* well-defined codes (Campbell et al., 2013).

*3. Coding process overview:*

- *Transcription & familiarization:* Coder 1 listened to all audio recordings, transcribed them *via* Descript, and performed open coding.
- *Sampling prior data for calibration:* Two random interviews from *SS’22* and two of the longest interviews from *WS’21* and *WS’22* were selected and coded separately by both coders.
- *Immersion & merging:* Coder 2 listened to select audio recordings (Bird, 2005) for thorough immersion, and we merged each coder’s initial codes (~70–80) to resolve naming conflicts.
- *Refinement rounds:* After two isolated refinement rounds, ICR remained below 0.70, prompting further adjustments in code definitions; a third iteration achieved ICR > 0.72.
- *Final consolidation:* In this final iteration, multiple fine-grained or duplicative codes were merged, while broad or ambiguous codes were split into more precise categories. Coder 2 verified which quotes best exemplified each refined code, and any remaining discrepancies were resolved through a negotiated agreement (Campbell et al., 2013).
- *Full application:* Once the scheme was stabilized, Coder 1 applied it to the remaining four SS’22, ten WS’21, and five WS’22 transcripts.

*4. ICR Computation (Krippendorff’s Alpha):*

We exported the coding assignments into a CSV file, ensuring a nominal scale representation suitable for the *K-Alpha* online calculator tool (Marzi, Balzano & Marchiori, 2024). Three iterative refinements achieved *Krippendorff’s α > 0.72*, indicating substantial reliability (Hayes & Krippendorff, 2007). Any remaining discrepancies persisting after the third round were reconciled *via* negotiated agreement.

#### Cross-study thematic integration and re-coding previous data

To integrate new findings with prior interview data (*WS’21*: 12 interviews; *WS’22*: 7 interviews), we selected two additional interviews from each past dataset and re-coded them using the newly refined framework. A further check yielded *α > 0.70*, confirming coding reliability. Next, Coder 1 re-coded all remaining prior interviews. Upon cross-analytical consensus discussions, some original themes were merged, re-labeled, or dropped if unsupported. For example:

- “Attention and focus deficits” (from WS’21) folded into *“Context switching & other factors”* or *“Mixed perceptions of time limits.”*
- *“Mentorship & Helping”* was separated from general “Feedback/Teaching” (WS’21 & WS’22) based on the more explicit mentor-like behaviors identified in SS’22.

#### Themes and saturation

Each study reached saturation within its respective scope and interview guide (Guest, Bunce & Johnson, 2006), yet the protocols evolved over time to address novel dimensions of pair programming. Consequently, the SS’22 study was deemed saturated after six interviews, surfacing two new themes—Theme 9 (“Perceived Task Difficulty & Project Stages”) and Theme 10 (“Context Switching & Other Factors”). Although we designed tasks to have uniform complexity, participants’ subjective perceptions varied. Some found certain tasks easier or harder, noting how pair programming reduced perceived difficulty or benefited particular project stages. Furthermore, context switching was addressed through an expanded set of interview questions.

Following these refinements, the final thematic map contained 12 overarching themes, each representing a facet of participants’ psychological, social, or technical experiences. These themes are reported in detail in the next Empirical Findings section.

## Empirical findings

In this section, we detail the results of our quantitative and qualitative analyses, which were drawn from multiple quasi-experimental studies (*WS’21*, *SS’22*, and *WS’22*). We begin by describing participant demographics and basic data characteristics, followed by descriptive statistics of the Big Five traits and the main findings from our linear mixed-effects (LME) models. Afterward, we summarize the thematic insights from 25 qualitative interviews.

### Quantitative results

*Participant demographics:* A total of 66 participants took part in the *WS’21* (*n* = 40) and *SS’22* (*n* = 26) studies combined. The sample comprised Gen-Z undergraduates from advanced software engineering courses, with 60 participants identifying as male and six as female. On average, participants reported 2.21 years of experience (SD = 2.31), wherein academic experience was counted at half-weight relative to industrial experience.

*Rationale for linear mixed-effects modeling:* We employed LME models to accommodate our within-subject, repeated-measures design (up to 18 programming rounds per participant) and to test interaction effects between Role (Pilot, Navigator, Solo) and continuous or categorical personality variables. This approach (1) accounts for individual baseline differences in intrinsic motivation *via* random intercepts, (2) manages missing data (*i.e*., four absences due to illness) more flexibly than repeated-measures ANOVA, and (3) allows complex interactions (Role × Big Five or Role × PersonalityCluster) to be captured in a single unified framework.

*Descriptive statistics for the Big Five Traits:* We first examined participants’ aggregated Big Five Inventory (BFI-10) scores (*n* = 66). Table 1 summarizes the mean, standard deviation, median, range (min/max), skewness, kurtosis, and Shapiro–Wilk *p*-values for each trait (Openness, Conscientiousness, Extraversion, Agreeableness, Neuroticism). Although Openness and Conscientiousness slightly violated normality (*p* < 0.05), we retained all traits in their original form given the robustness of LME models to moderate normality violations and our focus on repeated-measures data.

**Table 1:** Descriptive statistics of average Big Five trait scores (WS’21 + SS’22, *n* = 66).

Trait	Mean (SD)	Median	Min	Max	Skew	Kurtosis	Shapiro–Wilk (*p*)
Openness	3.55 (0.96)	3.67	1.17	5.00	−0.35	−0.69	0.0462
Conscientiousness	3.02 (0.81)	2.83	1.50	5.00	0.84	0.21	0.00052
Extraversion	2.89 (0.92)	3.00	1.00	5.00	0.05	−0.70	0.5514
Agreeableness	3.27 (0.77)	3.33	1.67	4.67	−0.24	−0.64	0.1408
Neuroticism	2.93 (0.98)	2.83	1.17	5.00	0.28	−0.73	0.1634

**Note:**

Scores range from 1 to 5, with higher values indicating stronger expression of each trait. A Shapiro–Wilk *p* < 0.05 suggests mild deviations from normality. Skew and kurtosis values between –1 and +1 typically indicate moderate normality.

#### Overall role effects (H1 & H1-Cor)

To assess whether intrinsic motivation varied by Role (H1) and remained stable within individuals (H1-Cor), we fit a linear mixed-effects model with Role (Pilot, Navigator, Solo) as a fixed effect and Participant as a random intercept, accounting for repeated measures. *Post-hoc* Tukey-adjusted comparisons (Table 2) showed a clear hierarchy of means: Pilot > Navigator > Solo. Specifically, Pilot was significantly more motivating than Solo (estimate ≈ +1.60, *p* < 0.0001), and Pilot also surpassed Navigator (estimate ≈ +0.56, *p* = 0.0328). This pattern remained consistent across all experimental rounds, indicating stable role-based motivation differences (supporting H1 & H1-Cor).

**Table 2:** LME interaction model role effects. Pairwise role comparisons from the “interaction” LME model (IntrinsicMotivation ~ Role * (B5_O + B5_C + B5_E + B5_A + B5_N), 66 participants, 1,092 total observations.

Contrast	Mean difference (7–35 scale)				Cohen’s *d*			
	Estimate (SE)	95% CI (Diff)	df	*p*-value	*d* (SE)	95% CI (*d*)	df	*p*-value
Pilot–Solo	1.604 (0.218)	[1.09 to 2.11]	1,014	<0.0001	0.576 (0.0793)	[0.4173–0.734]	60	<0.0001
Pilot–Navigator	0.562 (0.224)	[0.04–1.09]	1,014	0.0328	0.202 (0.0805)	[0.0407–0.363]	60	0.0150
Solo–Navigator	–1.042 (0.218)	[−1.55 to −0.53]	1,014	<0.0001	−0.374 (0.0787)	[−0.5316 to −0.217]	60	<0.0001

**Notes:**

(1) Intrinsic motivation is measured on a 7–35 scale. (2) A positive mean difference means the first role (*e.g*., Pilot) is higher than the second (Solo). (3) *p*-values for mean differences are Tukey-adjusted; df = 1,014 reflects the model’s denominator *df* under repeated-measures LME with many (1,092) observations. (4) Cohen’s *d* is computed from the residual SD (σ ≈ 2.785); df = 60 arises from emmeans handling repeated-measures factors in pairwise contrasts.

*Interpretation:* Participants generally found Pilot the most motivating role, Navigator intermediate, and Solo the least. This satisfies H1 (role differences exist) and shows stability over repeated rounds (H1-Cor).

#### Model fit and variance

We computed marginal and conditional *R*^*2*^ (Nakagawa & Schielzeth, 2013) to assess how much variance in intrinsic motivation was explained by fixed effects (Role, Big Five traits) versus random intercepts (Participant). In the Role × Big Five interaction model, the marginal *R*^*2*^ was approximately 0.11, whereas the conditional *R*^*2*^ rose to ~0.42–indicating that participant-level differences add ~31% more explained variance. A similar pattern emerged for the simpler Role *+* Big Five model (marginal *R*^*2*^ ~0.085; conditional *R*^*2*^ ~0.39). Hence, while Role and Personality account for a meaningful portion of intrinsic motivation, about half of the variance remains unexplained. In the *Discussion*, we elaborate on potential situational and measurement factors.

Supplemental Material S1 provides additional distributional and diagnostic plots, including *Q–Q* and residual-*vs*.-fitted checks that confirm the model residuals are approximately normal and exhibit no notable heteroscedasticity, supporting the appropriateness of our LME assumptions. That material also includes interaction predictions for Openness, illustrating that individuals at +1 SD in Openness show a stronger preference for the Pilot role, while Solo appears less demotivating for those at −1 SD.

#### Interaction effects of Big Five traits and personality clusters (H2–H6)

To explore how personality modifies these role preferences, we ran *two* interaction models:

1. *Role × Big Five:* Treating each trait (O, C, E, A, N) as a continuous predictor.
2. *Role × PersonalityCluster:* Assigning participants to whichever trait they scored highest on (Openness, Conscientiousness, Extraversion, Agreeableness, Neuroticism).

Table 3 merges these core results, focusing on the key comparisons of Pilot *vs*. Solo, Navigator *vs*. Solo, Navigator *vs*. Pilot, and the “Cluster Insights” (Role differences within each dominant-trait cluster). We interpret these results in light of H2 (distinct personality configurations exist), H3 (role preferences differ by personality cluster), and H4–H6 (trait-specific hypotheses).

**Table 3:** Summary of interaction effects in Role × Big Five and Role × PersonalityCluster models.

Trait	Cluster interaction insights	Big Five interaction–pilot *vs*. solo	Big Five interaction– navigator *vs*. solo	Big Five interaction– navigator *vs*. pilot
Openness	Positive for navigators (+1.02, *p* = 0.1706)	+0.53, *p* = 0.0254	+0.11, *p* = 0.6592	−0.42, *p* = 0.0808
Conscientiousness	Significant for solo programmers (+1.93, *p* = 0.0044)	−0.42, *p* = 0.1464	−0.99, *p* = 0.0006	−0.58, *p* = 0.0544
Extraversion	Significant for navigators (+1.58, *p* = 0.0403)	+0.26, *p* = 0.3471	+0.40, *p* = 0.1377	+0.14, *p* = 0.6003
Agreeableness	Significant for navigators (+2.22, *p* = 0.0002)	−0.04, *p* = 0.9021	+0.25, *p* = 0.3979	+0.29, *p* = 0.3376
Neuroticism	Significant for solo programmers (+1.87, *p* = 0.0033)	−0.78, *p* = 0.0005	−0.56, *p* = 0.0134	+0.22, *p* = 0.3489

**Notes:**

“Cluster Interaction Insights” describe the intrinsic motivation effect (7–35 scale) of being in a dominant-trait group (*e.g*., “Openness cluster”) on role preferences. “Big Five Interaction” columns report how each Big Five trait moderates role differences (Pilot *vs*. Solo, *etc*.) measured as a difference between chosen and baseline roles (7–35 scale). *p*-values are adjusted for multiple comparisons using Tukey’s method.

*Openness (H4):* Big Five interactions show a positive difference for Pilot *vs*. Solo among those high in Openness (+0.53, *p* = 0.0254), suggesting that creative, exploratory mindsets thrive in the Pilot role. The PersonalityCluster model hints at a smaller, non-significant boost for Navigator. Overall, high-openness developers are particularly motivated in Pilot roles.

*Conscientiousness:* A discrepancy arises: the Big Five model indicates Navigator is *less* motivating for conscientious developers (–0.99, *p* = 0.0006), whereas the cluster model suggests a positive effect for Solo (+1.93, *p* = 0.0044) and even a mild benefit for Navigator in some cases. Likely, conscientious individuals thrive in structured tasks (Solo), though cluster assignments can overemphasize a single trait at the expense of secondary dimensions.

*Extraversion (H5 part):* In the Big Five model, Extraversion yields small, non-significant improvements across roles. However, in the cluster model, *Extraversion* is significantly beneficial for Navigators (+1.58, *p* = 0.0403), implying that extraverted participants particularly enjoy guiding and communicating at a higher level.

*Agreeableness (H5 part):* Agreeable participants see negligible differences in the Big Five model, but in the cluster model, Navigator emerges as significantly more motivating (+2.22, *p* = 0.0002) than Solo. This aligns with the view that cooperation, feedback, and synergy are critical to the Navigator role.

*Neuroticism (H6):* The Big Five model shows that high-neuroticism developers are less motivated in Pilot or Navigator roles (compared to *Solo*). Correspondingly, the cluster model strongly supports a Solo preference (+1.87, *p* = 0.0033), indicating a preference for structured, solitary work that is less anxiety-provoking.

#### Integrating personality cluster insights with Big Five interactions

The PersonalityCluster model emphasizes each participant’s *dominant* trait, simplifying the interplay of multiple traits. Despite such reductionism, it largely confirms the Big Five interaction findings:

1. *Openness:* Both models agree that open-minded individuals excel in the creative, hands-on Pilot role.
2. *Extraversion & Agreeableness:* Both approaches highlight the advantage of sociability and cooperation for Navigator.
3. *Neuroticism:* Both suggest emotionally sensitive developers prefer the structured independence of Solo over dynamic, real-time collaboration.
4. *Conscientiousness:* The key discrepancy arises here; in the Big Five model, conscientious developers find highly dynamic roles (*e.g*., Navigator) less rewarding, but sometimes thrive in them if they can impose their own structure (per the dominant-trait cluster approach), priming the dyadic collaboration. In practice, conscientious programmers may excel anywhere but especially in *Solo* tasks, which allow meticulous focus.

Notably, *Pilot* had the highest overall motivational baseline (Table 2). Therefore, it is possible that the *Role × PersonalityCluster* interaction model detects fewer “positive boosts” for Pilot because many participants already rated Pilot favorably, leaving less variance to be explained by personality. Nonetheless, Pilot still emerges as a prime choice for those high in Openness.

In short, the cluster approach and the trait-interaction approach converge on most findings, reinforcing that personality-based assignments can amplify or attenuate the inherent motivational differences between Pilot, Navigator, and Solo. Nonetheless, real-world developers often exhibit multiple strong traits—an aspect that pure clustering may oversimplify. This dual-model approach thus provides complementary insights.

#### Individual variation and example

Beyond group-level effects, certain individuals displayed pronounced shifts in motivation across roles. For instance, a participant under the pseudo-ID “xtoj” experienced a *64.6%* increase in intrinsic motivation when moving from their least motivating role (Solo, mean = 13.7) to their most motivating role (Pilot, mean = 22.5). Such a case underscores the practical prospect of role alignment based on personality-driven preferences.

#### Summary of quantitative findings

In summary:

- *H1 & H1-Cor* were supported: Pilot proved most motivating, Solo least, and these differences remained consistent across repeated rounds.
- *H2 & H3* were validated by cluster-based results showing that Openness, Agreeableness, and Neuroticism meaningfully interacted with Role.
- *H4, H5, and H6:*
  - Openness (H4) strongly favors Pilot.
  - Extraversion & Agreeableness (H5) show the clearest benefits for Navigator.
  - Neuroticism (H6) finds Solo comparatively less demotivating.

Overall, the results support the value of personality-based role assignments. In very small entities (VSEs), careful role alignment may be–after validated generalizations and adaptations—especially critical given the high impact of each individual’s performance and motivation.

Having established a quantitative basis for personality—role alignments, we next explore how participants subjectively experienced pair programming through a thematic analysis of interview data.

### Qualitative results

The refined thematic analysis consolidates three data sets: *CIMPS–WS’21*, *EASE–WS’22*, and current SS’22, encompassing 25 interviews (12 + 7 + 6). While not all original codes or themes were retained, those that consistently appeared (or were illuminated by the new data) formed a cohesive final map of twelve overarching themes. As shown in Table 4, Themes 9 and 10 surfaced anew, capturing perceived task difficulty/project stages and context switching & other factors, respectively. Meanwhile, Themes 3, 11, and 12 were either newly discovered or substantially redefined during cross-analysis.

**Table 4:** Thematic system of twelve overarching themes reflecting undergraduates’ lived experiences with personality-based pair programming in three roles (pilot, navigator, solo). Twelve themes and sixty codes were derived from a cross-analysis of previous studies (WS’21 &WS’22) and the current interviews (SS’22) on personality-based pair programming, highlighting psychological, social, and technical dimensions of pair programming and offering insights into how participants’ personalities interact with role assignments to shape intrinsic motivation.

*Theme* & Origin	Representative quotes (ID)	Final stable codes (with pronounced participant references)
*1. Pairing Constellations* WS’22, Current	“Pairing with a familiar person allows you to anticipate their reactions and, therefore, be more honest with them, which is comfortable.” (P1) “If there was a system by which the partners were assigned to each other and stayed like that the whole semester, it would be nicer.” (P1) “If socially compatible, you can pair for longer.” (I2)	– Familiar Partners Facilitate Honesty (I2, P1, S2) – Similar Skill-Level and Work Pace Favored (P6, S3, I4) – Personality Extremes Lead to Conflict (S4, I1) – Long-Term Pair Stability Desired (P1, S5) – Social Compatibility Encourages Extended Collaboration (I2, P5)
*2. Feedback* WS’21, WS’22	“Feedback is important for everyone to progress in anything; whether negative or positive, it must be told.” (P4) “Sometimes he was really dominant, so I had to tell him ‘Yes, I can do this on my own.’” (P6) “Explain what he/she was doing to value me.” (I3)	– Valuing Different Opinions (I3, P2, S1) – Necessary for Continuous Improvement (P4, S3) – Corrective Guidance Builds Confidence (P6, I7) – Partner’s Feedback as Emotional Support (I5, P1) – Asking for Clarification Reinforces Understanding (S2, I9)
*3. Mentorship & Helping* Refined/New	“Of course, communication is an integral part of PP. During the conversation, you can discover errors, discuss the strategy, find a solution on which both agree and share knowledge in a great way.” (P2), “I felt like a teacher who could share his knowledge.” (S2)	– Teaching-Like Explaining (S2, I2) – Encouraging/Empowering Partner (P5, I6) – Emotional Support Through Helping (S3, P1) – Knowledge Transfer as Core Activity (P2, I4) – Mutual Problem-Solving Boosting Motivation (I5, S1)
*4. Soft & Social Skills* WS’21, WS’22	“Programming consists of two skills: programming something and asking questions perfectly. The latter is really difficult.” (P1) “PP was even better than on Discord or chat. You understand body gestures, eye signals, and so on.” (P5)	– Perfect Question Formulation (P1, I7), - Reading Nonverbal Cues (P5, S3), - Building Real-World Communication Skills (S2, I11) – Trust-Building Through Face-to-Face Interaction (P3, I9) – Difficulty in Asking for Help Gracefully (S4, I2)
*5. Psychological Aspects* WS’21, WS’22	“Almost everything can be solved in pairs, from programming to your emotional state.” (P2) “Roles and being observed push you out of your comfort zone.” (I5) “I enjoyed being part of something greater in this experiment.” (P7)	– Hawthorne Effect (I5, P1, S1) – Emotional Well-Being Improved by Pairing (S4, P2) – Personality-Role Alignment Eases Stress (P2, I3) – Self-Reflection Triggered by Collaborative Setting (P3, I10)
*6. Role-Specific Insights* WS’22, Current	“As the navigator, I felt like a leader. The whole situation was in my hands. It motivated me a lot!” (P5) “I liked that I could do my own research without someone watching in the solo role.” (P1) “Both roles check for a different type of errors.” (P2)	– Navigator Role as Leadership (P5, S2) – Pilot Focuses on Implementation Details (I6, P4) – Solo Role Allows Independent Exploration (P1, S3) – Responsibility Varies by Role (S6, I2) – Different Roles Offer Diverse Cognitive Workloads (I8, P3)
*7. Time Constraints & Progressivity* WS’21, Current	“I think the time limit was good because when not sitting at the computer for long, I lose focus. (…) Not having the same amount of seat time could spark a sense of unfairness or jealousy.” (P1) “Timer is confusing because you leave your job unfinished, and now you have to take on another role.” (P5)	– Mixed Perceptions of Time Limits (P5, S2) – Efficiency Gains from Deadlines (I9, S3) – Stress from Incomplete Tasks When Rotating (P1, S4) – Fairness Concerns Over Unequal Seat Time (P1, P6, I11) – Preference for Rotating After Completion (I3, S5)
*8. Perfect Pair Programmer’s Traits* WS’22	“Be communicative, have quick learning ability, the tendency to display self-discipline, be calm, willing to compromise their interest with others, and emotionally stable.” (P2) “Should have respect for others and their thoughts, good communication skills, and ability to listen carefully.” (P3)	– High Extraversion for Better Interaction (P2, S1) – High Agreeableness to Avoid Conflict (I2, P5) – Emotional Stability Reduces Tension (S3, I1) – Open-Mindedness Fosters Creativity (S2, P4) – Good Social Skills Enhance Trust & Rapport (P3, I6)
9*. Perceived Task Difficulty & Project Stages (New)	“In my case, I was three times more productive in pairing than when doing the same task at home.” (P3) “It was fun, you never feel lost.” (S2), “All tasks seem easier.” (P2) “I find the start of a project very good (for pair programming) because in my opinion, when you start in pairs, nobody gets really lost.” (S3)	–Pairing Eases Perceived Complexity (S3, P2) – Difficulty Not a Key Factor If Paired (I1, S5) – Early Project Stages Benefit Most from Pairing (P4, I2) – Complex Steps Made More Manageable (S1, I3) – Uniform Task Design Still Felt Diversely by Individuals (P6)
10*. Context Switching & Other Factors (New)	“When they switched, their mindsets still remained in the last role” (S5), “Sometimes the partner would stop and not say anything. That was not pleasing.” (S2), “Personal health and life events impact productivity and collaboration.” (S4), “My personality depends on the current mood of the day.” (I11)	– Attention Problems in Role Switch (S5, I3) – Personal Issues Affecting Performance (P2, S4) – External Stressors (Exams, Mood) Influence Focus (I7, P3) – Context Shifts Disrupt Flow (S2, I10) – Adjusting to Changing Conditions Over Sessions (P1, S6)
*11. Pair-Forming Processes* *(Refined/New)*	“Perhaps students could find the most fitting partner by having short ‘speed-dating’ programming sessions at the beginning of the course with many different partners and then choose.” (P1) “Give everyone a partner with a similar skill level.” (P3) “If socially compatible, you can pair for longer.” (S6)	– Personality-Based Matching (I2, P5, S3) – Equal Pace of Work as Criterion (P6, I1) – Avoiding High Neuroticism Pairs (S5, P4) – Using Psychometric Tools for Pair Selection (P1, I3) – Trial Sessions Before Final Pair Assignment (P1, S2, I8)
*12. Essential Software Engineering Improvements (Refined/New)*	“Both roles check for a different type of errors” (P2) “I like analyzing things and when I did not have to worry about coding, I had so much space in my brain, I had a different view and saw probably the best approach.” (P4) “I found ways I wouldn’t have found in the pilot role.” (S1)	– Early Defect Detection in Pairing (I8, P2) – Alternative Approaches from Navigator-Pilot Interplay (S1, P4), – Pleasure & Creativity in Joint Problem-Solving (S5, P3) – Incremental Learning of Best Practices (I4, P6) – Improved Overall Teamwork (S3, I2)

**Notes:**

*Legend:* “PP” stands for “pair programming”. (*) Themes 9 and 10 emerged *anew* from the current dataset. Themes *3, 11, and 12* were re-defined or discovered through cross-analysis with older data. All quotes are retained *verbatim*. Codes reflect the *final refined set* after achieving *Krippendorff’s alpha* > 0.72. Participant references are: *I#* (WS’21: I1–I12), *P#* (WS’22: P1–P7), *S#* (current SS’22: S1–S6). Many original codes/themes were merged, split, or removed if not consistently supported; for instance, multiple time-related codes were merged under “Mixed perceptions of time limits” (Theme 7), and trait-specific conflict replaced the single broad code “Personality mismatch”. Only the most illustrative quotes are presented in the table to represent each theme concisely; the displayed codes are not limited to those quotes but stem from a comprehensive analysis of all interview transcripts.

#### Narrative of themes

While all twelve themes are noteworthy, the following discussion highlights how the final mapping extends or refines earlier findings:

- *Pairing constellations (Theme 1)*. This theme remains among the most frequently mentioned. Participants emphasized that social compatibility and similar skill/work pace led to more honest communication, new friendships, and longer productive sessions. Conversely, extremes in certain traits could create tension, reflecting the interplay of personality and social alignment.
- *Feedback (Theme 2) & mentorship & helping (Theme 3)*. Previously, feedback and teaching codes were often blended under a single umbrella. The new dataset revealed an emergent “mentor-like” role, prompting us to tease out a separate mentorship category. Feedback remains crucial, but participants also described deeper “I felt like a teacher” experiences that enhanced self-efficacy and knowledge sharing.
- *Soft & social skills (Theme 4) & psychological aspects (Theme 5)*. These categories reaffirm earlier insights—*e.g*., the Hawthorne effect for motivation, emotional well-being, and communication challenges—but the data also illustrate how some students found pair programming a method to practice real-world simulation skills in a more psychologically safe environment than professional settings.
- *Role-specific insights (Theme 6)*. Although identified previously, new interviews more strongly underscored the navigator as leadership aspect and how the solo role offered a mental “breather” for advanced coders. This nuance clarifies why certain traits—introversion and extraversion—might better align with specific roles.
- *Time constraints & progressivity (Theme 7)*. Participants were divided on strict time limits: some valued them for efficiency and fairness, while others were frustrated by unfinished tasks. The merging of multiple time-limit codes (*e.g*., “Need extra time,” “Rotate after finishing tasks”) into a single code, “Mixed perceptions of time limits,” acknowledges participants’ varied stances.
- *Perfect pair programmer’s traits (Theme 8)*. The new data and re-coding confirm earlier beliefs that high extraversion, agreeableness, and emotional stability are valued. However, the emphasis on “willing to compromise with others” also hints at a synergy between conscientiousness and adaptability in successful pair programmers.
- *Perceived task difficulty & project stages (Theme 9)*.* Newly articulated, it captures how participants found pair programming especially beneficial for complicated tasks or early project phases to avoid disorientation. Despite uniformly designed tasks, subjective difficulty varied, prompting some to note a boost in perceived productivity when working in pairs.
- *Context switching & other factors (Theme 10)*.* Also emerging anew, it highlights that personal health, external stress (exams), or abrupt role changes sometimes hinder focus. This clarifies the prior “attention and focus deficits” theme from *CIMPS–WS’21*, showing how context switching specifically triggers disruptions.
- *Pair-forming processes (Theme 11) & essential software engineering improvements (Theme 12)*. Both represent re-defined or newly recognized expansions:
  - The Pair-Forming Processes theme explores speed dating for matching, personality-based assignments, or skill balancing as ways to optimize pair dynamics.
  - Essential Software Engineering Improvements emphasizes early defect detection, synergy of differing roles, and a broader range of alternative solutions discovered through pairing.

Overall, the qualitative data corroborate our quantitative results and elaborate how personality alignment not only influences intrinsic motivation but shapes social dynamics, feedback quality, and coping strategies under time constraints or complex tasks.

## Personality-based pair programming artifacts

This section presents the latest iteration of our Role-Optimization Motivation Alignment (ROMA) framework and its application in very small entity (VSE) contexts. We describe how these artifacts expand on prior insights by mapping personality-based pair programming tasks onto both the *ISO/IEC 29110* Software Basic Profile’s Project Management (PM) and Agile Guidelines’ Software Implementation (SI) processes, which are suitable for developing non-critical software (ISO/IEC DIS 29110-5-1-2, 2023; Galván-Cruz et al., 2021; ISO/IEC DIS 29110-5-4, 2024). We then provide role assignment guidance, including how to incorporate AI assistance (Valový & Buchalcevova, 2023). We also briefly note the possibility of integrating a distributed pair programming application (Valovy & Buchalcevova, 2025) that operationalizes personality-based pairing and ensures secure, continuous data collection in VSE and small/home-office (SOHO) contexts. Finally, we conclude with practical considerations and disclaimers.

These artifacts are directly informed by our empirical findings that Openness aligns strongly with Pilot roles, Extraversion and Agreeableness correlate with Navigator preferences, and Neuroticism favors Solo tasks (see “Empirical Findings”). Consequently, we formulated seven tasks (T1–T7) that integrate these role–trait insights into ISO/IEC 29110 processes, offering a structured blueprint for VSEs to optimize pair programming roles and sustain higher intrinsic motivation.

### ROMA framework extension for VSEs

Building on the empirical results described in earlier sections, the *ROMA* framework aligns programming roles (Pilot, Navigator, Solo) with individual traits measured by a Big Five Inventory (*e.g*., BFI-10). Extending this approach to VSEs requires accounting for:

1. *Limited personnel and role rotation:* Fewer team members mean each individual’s motivational state has an outsized impact on overall productivity.
2. *Agile-friendly adaptations:* ISO/IEC 29110’s Agile Guidelines map directly to sprint-based events (Planning, Execution, Review, Retrospective), making it simpler to introduce pairing tasks and personality-based role allocations within standard agile ceremonies.
3. *Flexible, continuous improvement:* The goal is to iteratively refine how roles are assigned, so that VSE teams can adapt as the project, tasks, or team composition evolves.

#### Seven new tasks (T1–T7) for ISO/IEC 29110 Software Basic Profile and Agile Guidelines

To integrate personality-based pairing into the ISO/IEC 29110 Software Basic Profile and Agile Guidelines’s *PM* and *SI* processes, we propose the following *seven tasks*, mapped onto the relevant *PM.1–4* and *E1–E7* events:

*T1. Integrate personality assessment in hiring (PM.1)*

*– Objective*: Ensure new hires fit well with existing team dynamics and motivational needs.
*– Implementation*: Administer a Big Five assessment toward the end of recruitment; interpret results to guide role preferences and synergy with current staff.

*T2. Assess multidimensional work motivation (PM.2)*

*– Objective*: Track the baseline and periodic evolution of each team member’s motivation, *e.g*., with the multidimensional work motivation scale (MWMS; Gagné et al., 2015).
*– Implementation*: Conduct these assessments at major milestones or sprint boundaries to identify shifts in intrinsic or extrinsic motivators.

*T3. Define the Big Five Assessment Strategy (PM.2)*

*– Objective*: Formalize how and when Big Five assessments are conducted, ensuring ethical handling of personality data and clarifying how it informs role assignments.
*– Implementation*: Document procedures for administering BFI-10, safeguarding privacy, and mapping trait profiles to potential roles responsibly.

*T4. Pairing task (SI: E3, E4)*

*– Objective*: Introduce a dedicated “Pairing Task” into the sprint backlog and track and execute it like any other task during Sprint Execution (E4).
*– Implementation*: During Sprint Planning (E3), list pair programming sessions as formal tasks. Pair roles are selected based on personality alignment, but remain flexible if the project or personal circumstances change mid-sprint.

*T5. Measure intrinsic motivation impact (PM.3 & SI: E4, E7)*

*– Objective*: Evaluate how pairing and role assignments affect developers’ intrinsic motivation, typically using the Intrinsic Motivation Inventory (IMI).
*– Implementation*: Administer short IMI surveys after pairing sessions (E4) or longitudinal MWMS at sprint’s end (E7). Summarize findings in the project’s assessment artifacts.

*T6. Review and update pairing strategies (PM.3 & SI: E6, E7)*

*– Objective*: Continuously refine personality-based role assignments based on feedback from retrospectives and quantitative measures of motivation.
*– Implementation*: Combine sprint retrospective discussions (E7) with any relevant data from T5 and sprint reviews (E6), then revise the project plan and the next sprint’s Pairing Tasks.

*T7. Support future planning and knowledge transfer (PM.4)*

– *Objective*: Document lessons learned about personality-based pairing so that future projects or other VSE teams can benefit.
– *Implementation*: Include a final summary in the Project Closure (PM.4), focusing on best practices, pitfalls, and recommended improvements for future cycles.

#### Mapping to ISO/IEC 29110 PM and SI processes

**Figure 1:**
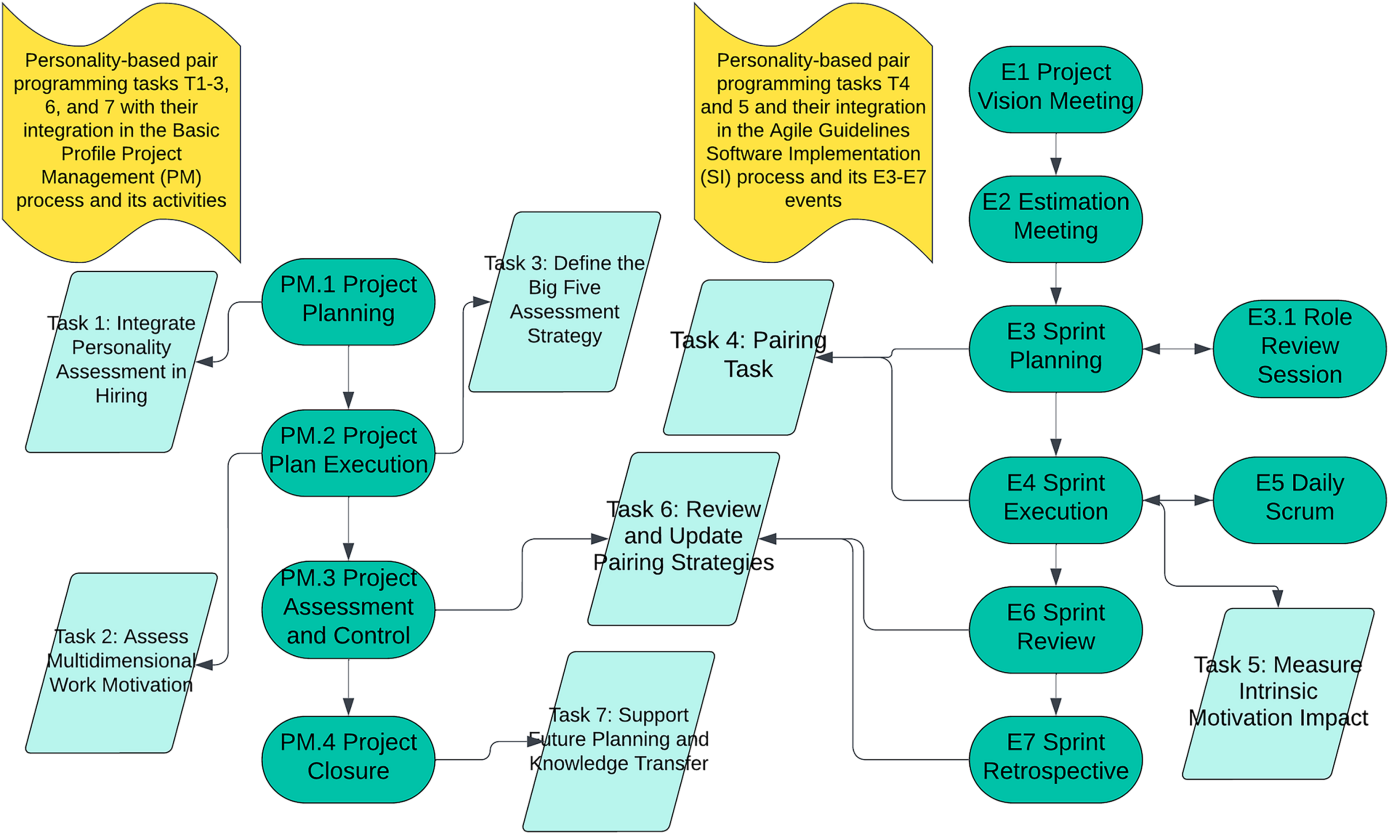
Personality-based pair programming Tasks T1–T7 for PM and SI processes of the ISO/IEC 29110 software basic profile and Agile guidelines. Illustration of how the seven personality‐based pair programming tasks (T1–T7) are integrated into the ISO/IEC 29110 Basic Profile Project Management (*PM*) processes (left) and the Agile Guidelines Software Implementation (*SI*) events (right). Teal ovals PM.1–PM.4 represent the four PM process stages, while E1–E7 indicate the SI events; light‐green rectangles label the personality‐based tasks T1–T7 and their placement within each stage or event. This diagram shows how to incorporate personality assessments, role review sessions, motivation tracking, and iterative pairing strategies into a typical sprint‐based workflow in Very Small Entities (VSEs).

Figure 1 illustrates how these seven tasks integrate seamlessly into *ISO/IEC 29110*:

1. *PM.1 Project Planning:* T1 and T3 help define the role assignments before coding begins.
2. *PM.2 Project Plan Execution:* T2 remains ongoing to track motivation changes.
3. *PM.3 Project Assessment & Control:* T5 and T6 measure how well pair programming is working and facilitate adjustments.
4. *PM.4 Project Closure:* T7 compiles the final lessons learned.
5. *SI (E1–E7 events):* T4 instantiates pair programming in the sprint backlog (E3, E4), T5 collects daily/weekly motivation data (E4, E7), and T6 uses sprint reviews and retrospectives (E6, E7) to refine approaches.

By embedding these tasks into the existing PM and SI activities, VSE teams can gradually adopt personality-based pairing without overhauling their entire software process.

### Role assignment guidance and AI optimizations

To facilitate *role optimization for motivational alignment (ROMA)* in both educational and professional settings, we recommend the following procedure:

1. *Administer a Big Five Inventory (e.g., BFI-10)* to each team member (T1, T3 in our tasks).
2. *Identify dominant traits:* Determine which trait(s) (Openness, Conscientiousness, Extraversion, Agreeableness, Neuroticism) stand out as relatively high.
3. *Map the trait(s) to a preferred role:* If Openness is high, consider the *Pilot* role; if Extraversion and Agreeableness predominate, Navigator may be best; if Neuroticism and/or Introversion is pronounced, Solo might be more comfortable.
4. *Account for secondary traits:* If no single trait clearly dominates, use either (a) a cluster approach or (b) a flexible “trial” period, rotating roles to see where each individual thrives.
5. *Incorporate AI insights* (Valový & Buchalcevova, 2023): The psychological effects of AI-assisted tools (*e.g*., GitHub Copilot or ChatGPT) may differ across roles. Tailor AI usage to each personality type and role preference, as summarized in Table 5 below.

**Table 5:** Personality–based pair programming optimization (including AI-assisted roles).

Personality traits & preferred role	Key characteristics	AI optimization (Valový & Buchalcevova, 2023)	Motivational impact	AI challenges (Valový & Buchalcevova, 2023)
Openness → Pilot (*Curiosity*)	Creative ideation, eager to explore novel solutions	AI as *brainstorming partner*, rapid code completion (*e.g*., Copilot)	Enhances autonomy & innovation; aligns with internal locus of control	Risk of over-reliance on AI; skill depth may decline if too reliant
Extraversion & Agreeableness → Navigator (*Interaction*)	Collaborative, team-oriented; guides partner through coding tasks	AI-assisted code review, identifies logical gaps, suggests alternatives	Encourages competence *via* structured oversight; supports group cohesion	Trusting AI suggestions without undermining human input; balancing opinions
Neuroticism & Introversion → Solo (*Reflection*)	Independent, detail-focused; thrives on structured tasks	AI handles repetitive searches, *frees cognitive load* for complex problems	Increases satisfaction by minimizing mundane tasks; supports self-paced learning	Potential isolation; must balance autonomy with AI input; risk of less peer feedback

**Notes:**

***How to Use the Table:*** *Matching Traits to Roles:* If an individual scores highest in Openness, encourage them to try the *Pilot* role first, possibly assisted by AI brainstorming. *Refining Role Assignments:* Where multiple traits are prominent, consider a short *trial period*—assign a role for an iteration or sprint, measure intrinsic motivation (T5), and reevaluate. *Addressing Challenges:* The “Challenges” column highlights where teams should be cautious. For example, in the *Navigator* role, ensure that AI suggestions do not override human communication and cooperation. *Scaling to VSEs:* In very small teams, a single developer may juggle multiple roles. Encourage short cycles of experimentation, ensuring that each role is assigned (or rotated) with these personality insights in mind. Integrating the Big Five–driven role optimization and assignment approach with AI assistance recommendations from Valový & Buchalcevova (2023).

By combining Big Five data with targeted AI usage, teams can better align each developer’s preferences, boosting intrinsic motivation and productivity. This complements the ROMA tasks (T1–T7), offering a role assignment blueprint adaptable to VSE contexts, agile ceremonies, and distributed or AI-assisted development scenarios. Further empirical validation in real-world projects is needed to confirm broader applicability and long-term outcomes.

### Practical considerations and disclaimers

*Practical adoption in VSEs:* Although each task is straightforward, privacy and trust remain key concerns. Teams should handle personality data ethically (*e.g*., anonymizing or restricting access). Additionally, tasks T2 and T5 require minimal but consistent effort to administer motivation surveys, ideally summarized at the close of each sprint or project phase.

*Controlled scope vs. real-world complexity:* Our framework arose from controlled studies (often with undergraduate participants), so its direct transfer to commercial VSE contexts demands further validation. Managers should remain aware of confounding variables like varying task complexity, skill diversity, and distributed settings that might magnify or dampen personality influences.

*Optional distributed pair programming application:* While beyond this study’s scope, we note that a blockchain-driven distributed pair programming tool (Valovy & Buchalcevova, 2025) can facilitate secure, privacy-friendly execution and data collection for T2–T6 in co-located and remote VSE or SOHO contexts. By embedding personality and motivation assessments within the tool, teams can seamlessly gather and analyze ongoing data for continuous improvement.

### Concluding remarks on the artifacts

The ROMA framework extension, the seven tasks (T1–T7), and role assignment guidance in Table 5 collectively offer a structured methodology for integrating personality-based pair programming into non-mission-critical VSE teams. By thoughtfully adapting ISO/IEC 29110 PM and SI processes, this approach helps small teams leverage individual differences to boost intrinsic motivation, potentially enhancing software quality and project outcomes. Although these insights stem from undergraduate participants, further professional validation is warranted to confirm their broader applicability. Future work may refine these artifacts as more VSEs trial personality-based pair programming in diverse technical and cultural settings, including remote or AI-assisted development.

## Discussion

This study investigated how personality-based role assignments in pair programming influence intrinsic motivation—particularly among Gen-Z undergraduates but with the intent to inform very small entities (VSEs), where each individual’s contribution can disproportionately impact project outcomes. Guided by *RQ1*, *RQ2*, and *RQ3*, we conducted a mixed-methods investigation combining LME-based quantitative analyses of two combined data sets (WS’21, SS’22) and qualitative thematic cross-analysis involving a third dataset (WS’22). By *triangulating across three studies*, we observed *consistent trait-role alignments* and motivational patterns, suggesting that *Big Five-informed* role optimizations can yield appreciable gains in intrinsic motivation.

### Key Findings for RQ1: personality-based motivational preferences

*RQ1* asked: *“What new understanding of personality-based motivational preferences for Pilot, Navigator, and Solo programming roles can be achieved?”*

Our LME results showed that:

- *Openness* aligns well with the Pilot role, highlighting that creative individuals are likely to enjoy and excel in hands-on, exploratory coding tasks (DeYoung, 2015).
- *Extraversion* and Agreeableness predispose individuals to Navigator roles, consistent with prior findings that interpersonal synergy and proactive communication (Williams & Kessler, 2003) benefit pair programming.
- *Neuroticism* aligns more comfortably with Solo work, suggesting that structured, lower-stress environments reduce anxiety and help individuals with higher negative affect remain motivated (Latham, 2012).

This significantly expands prior research (*e.g*., Hannay et al., 2009b) by showing that intrinsic motivation—rather than performance alone—is a key outcome of role-trait alignment. Some participants reported up to a 60–65% boost in intrinsic motivation when assigned to roles matching their personality traits. Although these gains require further validation in professional VSE contexts, they highlight a potentially powerful means of optimizing developer engagement in small teams.

### Socio-psychological and group-dynamic effects (RQ2)

*RQ2* targeted: *“What are the socio-psychological and group dynamic effects of pair programming?”*

From 25 interviews, we identified twelve overarching themes that reflect how personality-based pair programming fosters or impedes individual motivation and team collaboration. Illustrative examples include:

- *Pairing constellations:* Participants repeatedly noted that role-aligned social compatibility and similar skill/work pace reduce cognitive load and friction—resonating with findings by Sfetsos et al. (2009) on advantages of heterogeneous pairs, Zieris & Prechelt (2020b) on mental model alignment, and by Katira et al. (2004) on similar skill level and dissimilar personality type pairing compatibility.
- *Mentorship & helping:* This theme parallels the teaching and coaching dynamics observed by Williams et al. (2008), showing that constructive feedback loops enhance self-efficacy and mutual respect.
- *Context switching:* While enforced rotations promoted fairness and balanced team dynamics (Gallis, Arisholm & Dyba, 2003; Forsyth & Elliott, 1999), some participants felt mental carryovers from the previous role, complicating transitions between coding tasks, indicating that time slicing and rapid role changes can disrupt flow states unless carefully managed.

Overall, these social and psychological factors underscore that *how* roles are assigned and rotated can shape pair programming’s motivational outcomes as much as *which* roles are adopted.

### Practical implications—the ROMA framework in VSEs (RQ3)

*RQ3* asked: *“How should the ROMA framework, optimizing programming roles and their assignments, be utilized in VSEs?”*

Our final contribution proposes mapping the Role-Optimization Motivation Alignment (ROMA) framework to the *ISO/IEC 29110* Software Basic Profile and Agile Guidelines, capturing tasks T1–T7 to operationalize personality-driven pair programming. This structured extension directly responds to calls for lightweight, context-sensitive software processes in VSEs (O’Connor & Laporte, 2014). Specifically:

- *Tasks 1–3* integrate personality and motivation assessments in hiring and project planning.
- *Tasks 2 and 5* enable teams to detect motivation dips using simple, ongoing instruments (IMI, MWMS) and intervene swiftly.
- *Tasks 4–6* introduce iterative pairing tasks and frequent feedback loops in agile sprints (E3–E7), consistent with the continuous improvement ethos of agile methods (Beck et al., 2001).
- *Task 7* emphasizes lessons learned for future VSE endeavors, ensuring knowledge transfer beyond a single project cycle.

By tying personality alignment to standard VSE processes, we offer a structured, minimally disruptive approach for agile routines. References to AI-assisted and distributed pair programming (Valový & Buchalcevova, 2023, 2025) further suggest how these insights could be scaled or adapted to remote, technology-augmented settings. Although potential long-term benefits (*e.g*., retention, turnover reduction) remain hypothetical without dedicated field studies, our results strongly suggest that role—trait matching fosters higher immediate intrinsic motivation and basic psychological needs satisfaction, which could translate to such benefits over longer time frames (Ryan & Deci, 2017).

### Comparisons with related work

These findings complement earlier studies on pair programming and motivation. For instance, Arisholm et al. (2007) or Hannay et al. (2009a, 2009b) concluded that the effects of pair programming on performance are context-dependent, and our data indicate personality moderates those outcomes by shaping intrinsic motivation. Meanwhile, Aghaee et al. (2015) highlighted that intrinsically motivating role alignment must consider trait factors, which our personality-based approach addresses more systematically. Additionally, the attention to “time-slicing” and “unfinished tasks” parallels the need for pair programming guidelines (*e.g*., Williams et al., 2008). Our results reinforce that alignment can be boosted when role assignments reflect developers’ basic psychological needs, rather than a universal or random approach (Ryan & Deci, 2017).

### Limitations and generalizability

Several constraints limit the immediate generalizability of our study:

1. *Sample characteristics:* Although advanced undergraduates can approximate novice professionals (Falessi et al., 2018; Feldt et al., 2018), they still differ from experienced developers in real commercial environments. Our results must, therefore, be validated with a broader range of professional VSE teams.
2. *Controlled tasks & context:* While tasks were carefully designed to be comparable and actual to industry, real-world software projects often involve dynamic requirements, domain complexities, and team structures (Sjøberg et al., 2002).
3. *Personality measures:* We relied on the BFI-10, which is a concise instrument. Although it has acceptable validity, future studies might consider more detailed inventories to capture nuanced personality facets (Felipe et al., 2023).
4. *Focus on intrinsic motivation:* We primarily evaluated intrinsic motivation. While critical, other motivational constructs (extrinsic drivers, team climate, job satisfaction) may also be influential in professional contexts (Latham, 2012).
5. *AI tools:* Recommendations for AI usage are preliminary, based on emergent findings (Valový & Buchalcevova, 2023). Different AI tools or future evolutions of Large Language Models may yield varied psychological impacts.
6. *Partially untracked variables and unexplained variance:* Although our LME models explained up to ~42% of the variance in intrinsic motivation, a substantial portion remains unaccounted for. This residual variance may stem from untracked situational factors (*e.g*., group reconfigurations, day-to-day mood, cultural aspects), measurement noise, or the brevity of the BFI-10. Future work could incorporate richer personality instruments or control for these covariates—such as prior teamwork history or project complexity—to increase explanatory power.

#### Triangulation across three datasets and demographics

To enhance robustness, we cross-analyzed three triangulated datasets: WS’21, SS’22, and partial WS’22, observing stable trait–role alignments. In total, 66 participants contributed to the main quantitative analysis (WS’21 + SS’22), averaging 2.21 years of experience (counting academic experience at half), with six females and 60 males. While regionally limited, these samples offer cautious generalization to junior professionals with similar backgrounds. We addressed potential author bias through reflexive thematic analysis (Braun & Clarke, 2022) and maintained high inter-coder reliability (O’Connor & Joffe, 2020).

#### Future work

Given these limitations, several avenues for future research and development emerge:

1. *In situ evaluations:* Testing ROMA with professional VSE teams in multi-month sprints would clarify how role reassignments fare under real project scopes, shifting requirements, and potential distributed collaborations.
2. *Integration with different personality frameworks:* Beyond the Big Five, exploring other trait models (*e.g*., HEXACO) or combining interest inventories (*e.g*., RIASEC) might reveal deeper layers of role alignment.
3. *AI partner paradigms:* Investigating how advanced AI “co-pilot/navigator” tools might offset certain personality mismatches could refine or expand the ROMA framework’s utility, especially for developers high in Neuroticism or Introversion.
4. *Cultural and cross-organizational diversity:* Testing the ROMA framework in VSEs across various regions and organizational cultures would enhance the framework’s adaptability.
5. *Behavioral outcomes and software metrics:* Extending beyond motivational constructs, future studies could correlate personality-based role assignments with objective performance indicators (code quality, defect density, lead time) to quantify the overall impact on projects.

## Conclusion

By examining how personality-based programming role assignments shape intrinsic motivation, our mixed-methods study offers evidence that carefully matching Pilot, Navigator, and Solo roles to Big Five traits can measurably enhance developer engagement. While our data come from controlled academic contexts with Gen-Z undergraduates, the ROMA framework and tasks *T1–T7-*mapped to the ISO/IEC 29110 Software Basic Profile and Agile Guidelines—provide a structured blueprint for very small entities (VSEs) seeking to optimize limited human resources. Though potential long-term benefits, such as retention or reduced turnover, remain hypothetical without extended professional trials, the immediate motivational benefits observed here support further investigation. Ultimately, this work underscores the broader principle that software engineering success depends on understanding and accommodating people, their traits, and their motivations as much as on technical practices or tools.

## Supplemental Information

10.7717/peerj-cs.2774/supp-1 Supplemental Information 1 Experimental dataset of 26 participants from Summer semester 2022.

10.7717/peerj-cs.2774/supp-2 Supplemental Information 2 Combined dataset of 66 participants from Winter semester 2021 and Summer semester 2022.

10.7717/peerj-cs.2774/supp-3 Supplemental Information 3 Data Preparation and Initial Computations. This script:
1) Reads raw experiment data from the specified Excel file(s).
2) Preprocesses and factorizes Big Five and Role columns.
3) Creates per-session data files and aggregated Big Five averages.
4) Writes out three key files: # - Stats_…_Session_Data.xlsx # - Stats_…_B5Avg.xlsx # - Stats_…_Ready.xlsx

10.7717/peerj-cs.2774/supp-4 Supplemental Information 4 Linear Mixed‐Effects Modeling and Multi‐model Approach. This script:
1) Loads the combined dataset (Stats_WS2021+SS2022_Ready.xlsx).
2) Prepares the data for mixed-effects modeling (*e.g*., pivot to long).
3) Fits mixed-effects models with two different baseline roles (Pilot, Solo).
4) Produces a boxplot of IntrinsicMotivation by Role.
5) Saves model summaries and a PDF with diagnostic plots.

10.7717/peerj-cs.2774/supp-5 Supplemental Information 5 Post‐hoc Testing for Role‐based Comparisons. This script:
1) (Re)fits ‘model_interaction’ & ‘model_cluster’ if needed.
2) Performs *post-hoc* tests on Role-based & Cluster-based models.
3) Computes Cohen’s d for pairwise Role differences in the interaction model.
4) Outputs results to .txt files and optionally plots.

10.7717/peerj-cs.2774/supp-6 Supplemental Information 6 Big Five Descriptive Analyses. This script:
1) Reads the Big Five averages dataset (Stats_B5Avg).
2) Computes descriptive statistics (descriptives + Shapiro–Wilk).
3) Loads the main “Ready” dataset for demographic info.
4) Summarizes demographics.

10.7717/peerj-cs.2774/supp-7 Supplemental Information 7 Graphical diagnostics of intrinsic motivation by programming role.

10.7717/peerj-cs.2774/supp-8 Supplemental Information 8 Mixed-effects modeling output for Big Five fixed-effects with Solo baseline.

10.7717/peerj-cs.2774/supp-9 Supplemental Information 9 Mixed-effects modeling output for Big Five fixed-effects with Pilot baseline.

10.7717/peerj-cs.2774/supp-10 Supplemental Information 10 Mixed-effects modeling output for Big Five interactions with Solo baseline.

10.7717/peerj-cs.2774/supp-11 Supplemental Information 11 Mixed-effects modeling output for Big Five interactions with Pilot baseline.

10.7717/peerj-cs.2774/supp-12 Supplemental Information 12 Mixed-effects modeling output for PersonalityCluster interactions with Solo baseline.

10.7717/peerj-cs.2774/supp-13 Supplemental Information 13 Mixed-effects modeling output for PersonalityCluster interactions with Pilot baseline.

10.7717/peerj-cs.2774/supp-14 Supplemental Information 14 *Post-hoc* tests on Role × Big Five interaction model.

10.7717/peerj-cs.2774/supp-15 Supplemental Information 15 *Post-hoc* effect sizes on Role × Big Five interaction model.

10.7717/peerj-cs.2774/supp-16 Supplemental Information 16 *Post-hoc* tests on Role × PersonalityCluster interaction model.

10.7717/peerj-cs.2774/supp-17 Supplemental Information 17 The interview protocol listing the 25 questions used during the semi-structured interviews.

10.7717/peerj-cs.2774/supp-18 Supplemental Information 18 Experimental Questionnaire. The survey instrument administered during the quasi-experimental sessions.

## Additional Information and Declarations

### Data Availability

The following information was supplied regarding data availability:

The raw data and R scripts are available in the Supplemental Files.
