# Supplementary material for: Personality-based pair programming: toward intrinsic motivation alignment in very small entities
Source: PeerJ Comput Sci. 2025 Apr 1;11:e2774. doi: 10.7717/peerj-cs.2774 (PMC12190715; doi:10.7717/peerj-cs.2774)
Supplement: Supplemental Information 7 [file peerj-cs-11-2774-s007.pdf]

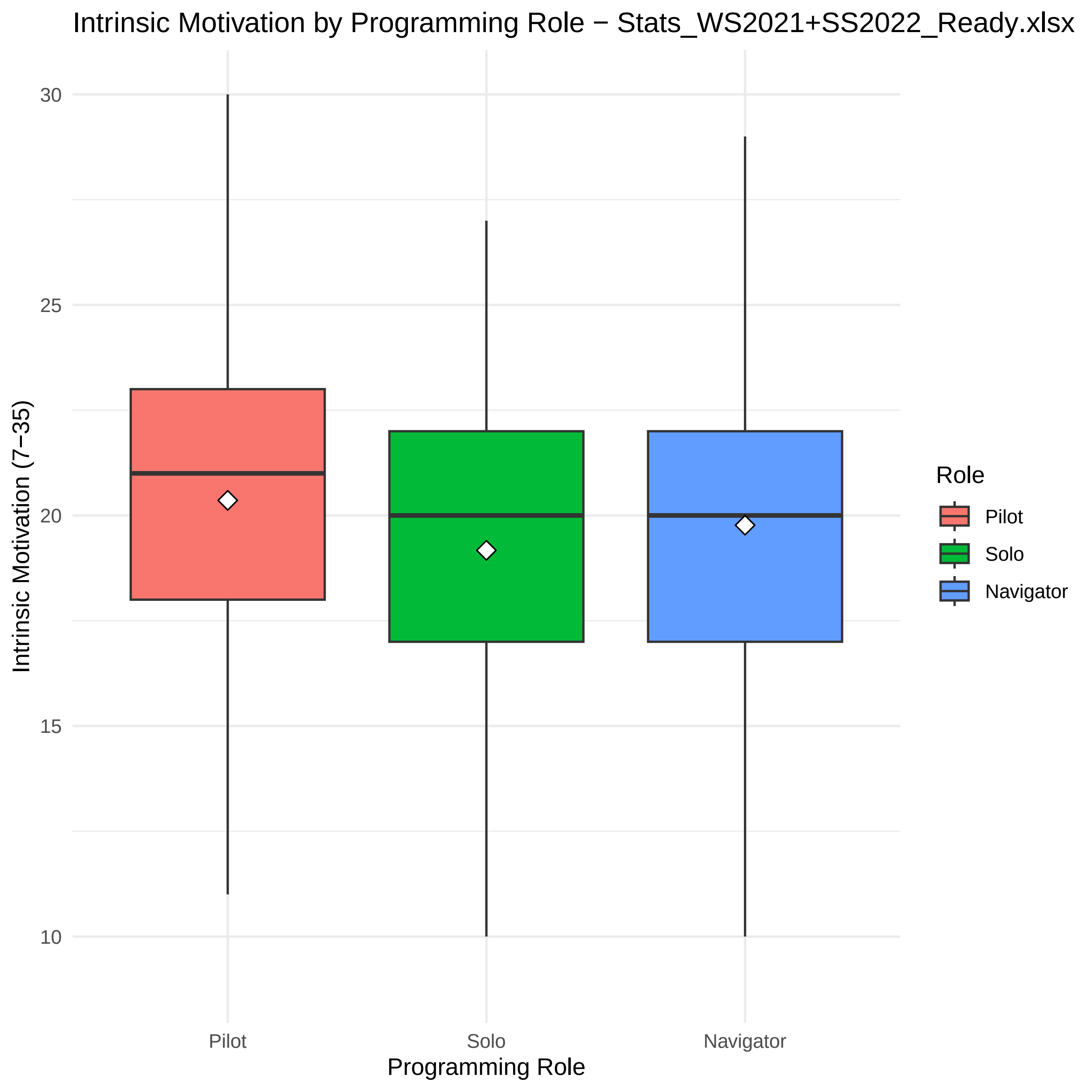

Intrinsic Motivation by Programming Role − Stats_WS2021+SS2022_Ready.xlsx
30
25
20
15
10
Intrinsic Motivation (7−35)
Pilot
Solo
Navigator
Programming Role
Role
Pilot
Solo
Navigator

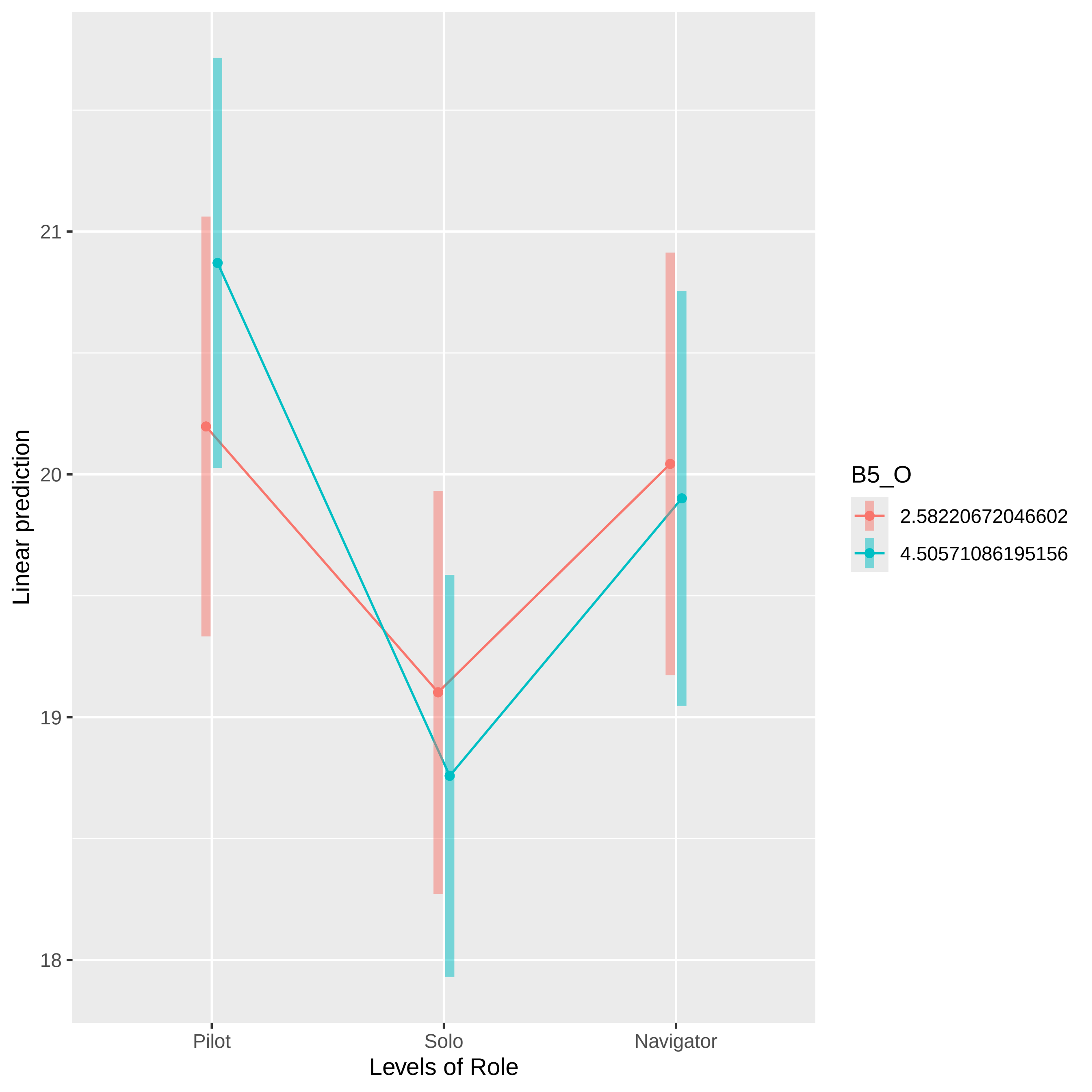

Linear prediction
21
20
19
18
Pilot
Solo
Navigator
Levels of Role
B5_O
2.58220672046602
4.50571086195156

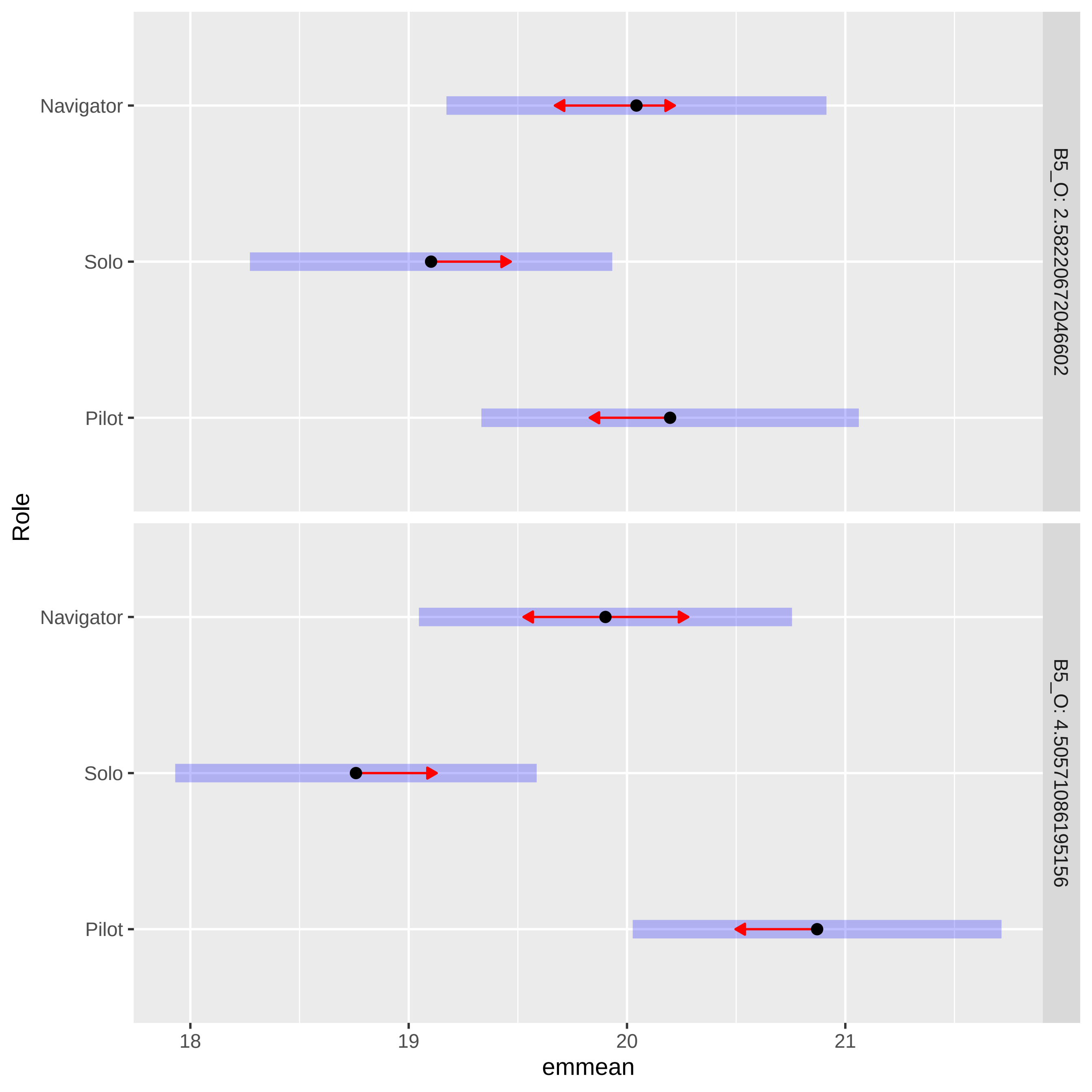

Navigator
Solo
Pilot
B5_O: 2.58220672046602
Navigator
Solo
Pilot
B5_O: 4.50571086195156
Role
18
19
20
21
emmean

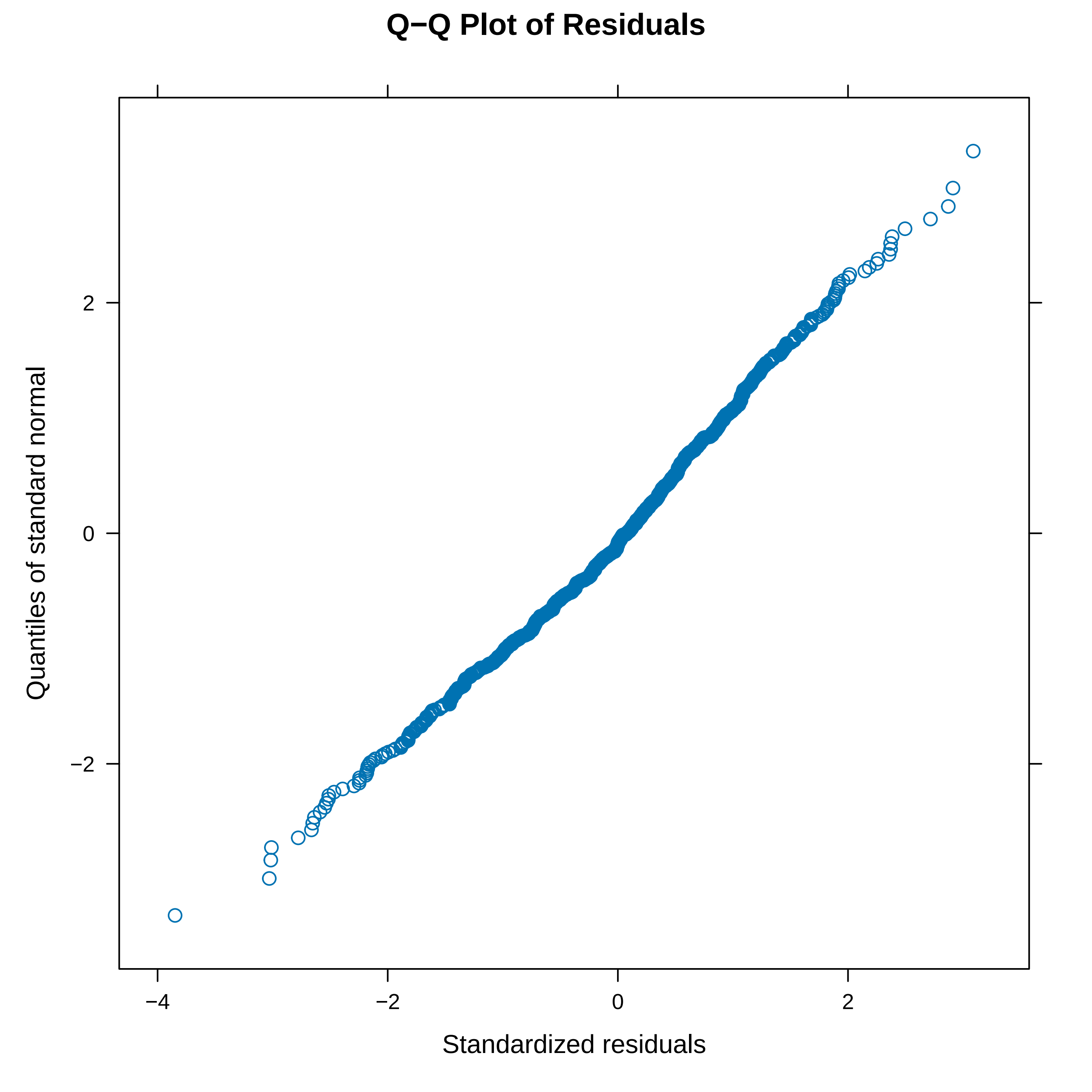
Q−Q Plot of Residuals
Quantiles of standard normal
Standardized residuals
−4
−2
0
2
−2
0
2

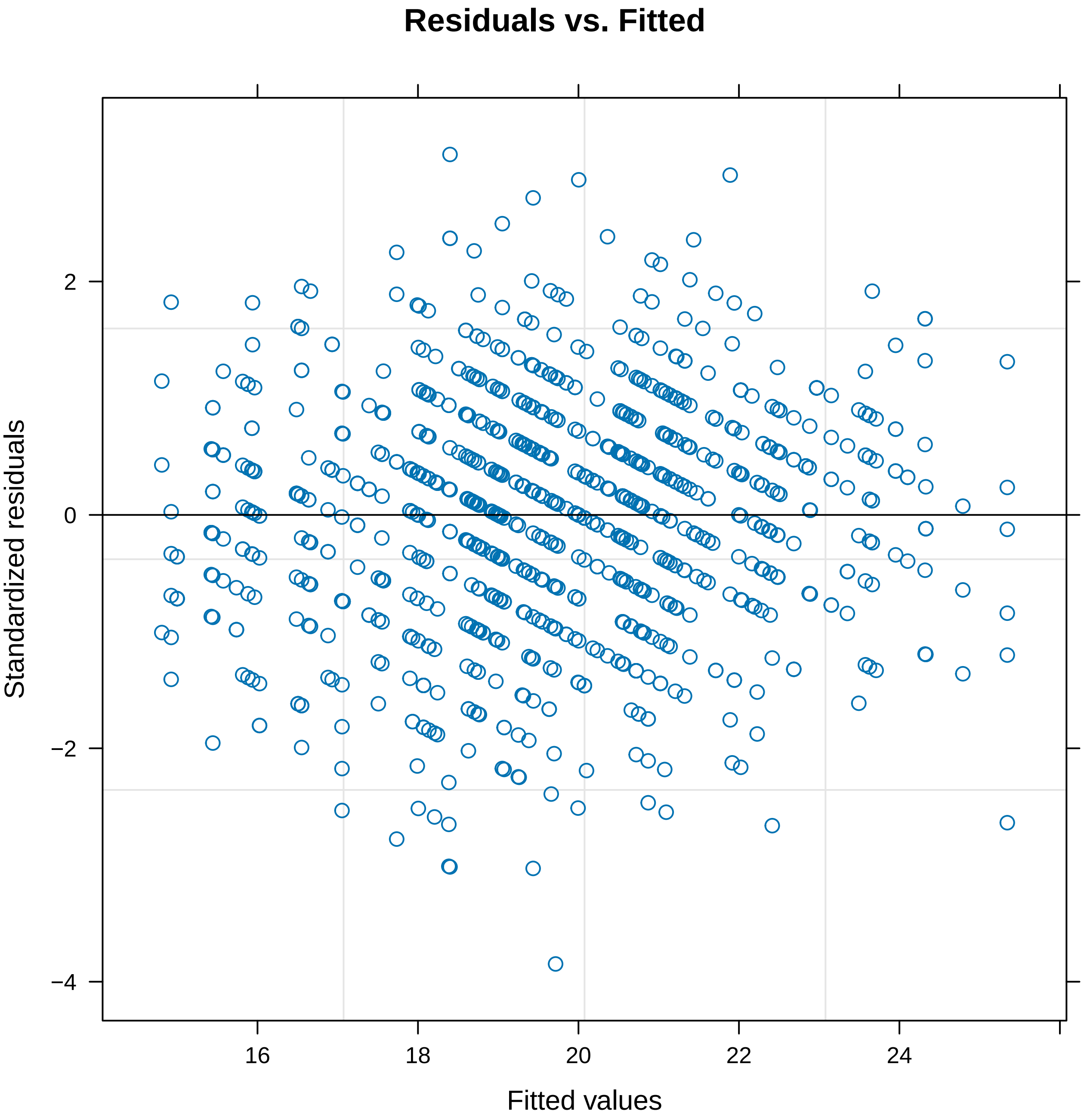
Residuals vs. Fitted
Standardized residuals
Fitted values
2
0
−2
−4
16
18
20
22
24
