# Supplementary material for: Personality-based pair programming: toward intrinsic motivation alignment in very small entities
Source: PeerJ Comput Sci. 2025 Apr 1;11:e2774. doi: 10.7717/peerj-cs.2774 (PMC12190715; doi:10.7717/peerj-cs.2774)
Supplement: Supplemental Information 17 [file peerj-cs-11-2774-s017.rtf]

Part 1: Career satisfaction.What does the career path you are pursuing look like?How satisfied are you with your choice of career?Part 2: Software engineering relationshipHow would you describe your relationship with programming and software engineering (in general)?How satisfied are you with software engineering courses at your university?Part 3: Experiment satisfactionHow would you describe your motivation to participate in scientific experiments (in general)?How satisfied were you with your participation in our experiments, specifically? If not - what change would you propose?How was this experience beneficial to you?How do you like Pair Programming?Part 4: Experiment Task satisfactionWas the nature of the tasks you were working on particularly affecting your motivation?Was it more motivating for you to work on the menu bar, animations, or team project?How would you rate the experience of working on a team project with your team members sitting around you?What impact did the amount of work completed have on your motivation?How did you manage to complete the tasks within the allotted time of 10 mins per each?Name the difficulties you encountered when switching roles with regard to picking up on the task where your partner has left?Were the tasks difficult for you? (On a scale of 1-10.)Were there any external factors affecting your motivation?Part 5: Partner satisfactionHow well did you know your partner up front?Who was doing most of the work?Was your partner more experienced than you?How well did your partner respect the frequent change of the roles?How would you describe the cooperation between you and your partner (during the experiment)?What feelings were you experiencing when your partner was navigating you?Name the pros and cons of being navigated vs navigating your partner.Part 6: Role satisfactionWhich role did you like the most (Pilot, Navigator, Solo)?How would you describe your satisfaction with being a Pilot during the experiment?How would you describe your satisfaction with being a Navigator during the experiment?How would you describe your satisfaction with being a Solo during the experiment?Part 7: PersonalityHow accurate do you think your personality test results are?Do you think your personality has had some effect on whether you liked being a Pilot, Navigator, or Solo?Part 8: Last RemarksDo you have anything to add? Final comments?Thank you!
