# Supplementary material for: Personality-based pair programming: toward intrinsic motivation alignment in very small entities
Source: PeerJ Comput Sci. 2025 Apr 1;11:e2774. doi: 10.7717/peerj-cs.2774 (PMC12190715; doi:10.7717/peerj-cs.2774)
Supplement: Supplemental Information 18 — The survey instrument administered during the quasi-experimental sessions. [file peerj-cs-11-2774-s018.pdf]

Experiment - 1.4.2022 (IGA-34/2021)

Dear University Student of Software Engineering Course,

Procedure:
If you agree to be in this study, you will be asked to do the following:
1. Perform 6 software engineering tasks, each taking 10 minutes.
2. Report your intrinsic motivation associated with the activity.
3. Answer questions about your personality.

The total time required to complete the study should be approximately 70 minutes. You will receive extra credit in your software engineering course for participating and have your psychometric results delivered to you in paper form, should you choose so.

Benefits/Risks to Participant:
Participants will learn about the empirical methodologies of and will help contribute to the body of knowledge in software engineering and psychology. Risks include any discomfort you may feel while performing the tasks and rating your motivation or responding to personality measurement questions.

Voluntary Nature of the Study/Confidentiality:
Your participation in this study is entirely voluntary and you may refuse to complete the study at any point during the experiment or refuse to answer any questions with which you are uncomfortable. You may also stop at any time and ask the researcher any questions you may have. Your name will never be connected to your results or to your responses on the questionnaires; instead, a number will be used for identification purposes. Information that would make it possible to identify you or any other participant will never be included in any sort of report. The data will be accessible only to those working on the project.

Thank You for participating in the Research on motivation management in software development teams *(IGA-34/2021)*.

Marcel Valový and Alena Buchalcevová
Information Technologies Department
Prague University of Economics and Business
xvalm00@vse.cz

* Required

1. I have read the Consent Form 1.4.2022 and I: *

- ◯ Agree to Participate
- ◯ Do not agree to Participate

2. I have x year(s) of experience: *

The value must be a number

3. I am a: *

- ◯ Male
- ◯ Female
- ◯ Prefer not to say

4. **Big Five Inventory-10 (Rammstedt & John, 2007)**
This question should take one minute or less.

Instructions: How well do the following statements describe your personality?
The stem is "I see myself as someone who..." *

	Disagree strongly	Disagree a little
1. ...is reserved.	◯	◯
2. ...is generally trusting.	◯	◯
3. ...tends to be lazy.	◯	◯
4. ...is relaxed, handles stress well.	◯	◯
5. ...has few artistic interests.	◯	◯
6. ...is outgoing, sociable.	◯	◯
7. ...tends to find fault with others.	◯	◯
8. ...does a thorough job.	◯	◯
9. ...gets nervous easily.	◯	◯
10. ...has an active imagination.	◯	◯

5. **Intrinsic Motivation Inventory (The Self-determination Framework, Ryan & Deci, 2015)**
Interest/Enjoyment - 10 *

	Disagree strongly	Disag
1. I enjoyed doing this activity very much	◯	
2. This activity was fun to do	◯	
3. I thought this was a boring activity	◯	
4. This activity did not hold my attention at all	◯	
5. I would describe this activity as very interesting	◯	
6. I thought this activity was quite enjoyable	◯	
7. While I was doing this activity, I was thinking about how much I enjoyed it	◯	
I was a: Pilot = Option 1, Solo = Option 3, Navigator = Option 5	◯	

6. Interest/Enjoyment - 20 *

	Disagree strongly	Disag
1. I enjoyed doing this activity very much	○	
2. This activity was fun to do	○	
3. I thought this was a boring activity	○	
4. This activity did not hold my attention at all	○	
5. I would describe this activity as very interesting	○	
6. I thought this activity was quite enjoyable	○	
7. While I was doing this activity, I was thinking about how much I enjoyed it	○	
I was a: Pilot = Option 1, Solo = Option 3, Navigator = Option 5	○	

7. Interest/Enjoyment - 30 *

	Disagree strongly	Disag
1. I enjoyed doing this activity very much	◯	
2. This activity was fun to do	◯	
3. I thought this was a boring activity	◯	
4. This activity did not hold my attention at all	◯	
5. I would describe this activity as very interesting	◯	
6. I thought this activity was quite enjoyable	◯	
7. While I was doing this activity, I was thinking about how much I enjoyed it	◯	
I was a: Pilot = Option 1, Solo = Option 3, Navigator = Option 5	◯	

8. Interest/Enjoyment - 40 *

	Disagree strongly	Disag
1. I enjoyed doing this activity very much	○	
2. This activity was fun to do	○	
3. I thought this was a boring activity	○	
4. This activity did not hold my attention at all	○	
5. I would describe this activity as very interesting	○	
6. I thought this activity was quite enjoyable	○	
7. While I was doing this activity, I was thinking about how much I enjoyed it	○	
I was a: Pilot = Option 1, Solo = Option 3, Navigator = Option 5	○	

9. Interest/Enjoyment - 50 *

	Disagree strongly	Disag
1. I enjoyed doing this activity very much	◯	
2. This activity was fun to do	◯	
3. I thought this was a boring activity	◯	
4. This activity did not hold my attention at all	◯	
5. I would describe this activity as very interesting	◯	
6. I thought this activity was quite enjoyable	◯	
7. While I was doing this activity, I was thinking about how much I enjoyed it	◯	
I was a: Pilot = Option 1, Solo = Option 3, Navigator = Option 5	◯	

10. Interest/Enjoyment - 60 *

	Disagree strongly	Disag
1. I enjoyed doing this activity very much	◯	
2. This activity was fun to do	◯	
3. I thought this was a boring activity	◯	
4. This activity did not hold my attention at all	◯	
5. I would describe this activity as very interesting	◯	
6. I thought this activity was quite enjoyable	◯	
7. While I was doing this activity, I was thinking about how much I enjoyed it	◯	
I was a: Pilot = Option 1, Solo = Option 3, Navigator = Option 5	◯	

11. I want my psychometric results (in a paper or electronic confidential form): *

◯ Yes

◯ No

12. My initials are: *

This content is neither created nor endorsed by Microsoft. The data you submit will be sent to the form owner.

Microsoft Forms
